# mTORC1 regulates high levels of protein synthesis in retinal ganglion cells of adult mice

**DOI:** 10.1016/j.jbc.2022.101944

**Published:** 2022-04-18

**Authors:** Patrice E. Fort, Mandy K. Losiewicz, Lynda Elghazi, Dejuan Kong, Corentin Cras-Méneur, Diane C. Fingar, Scot R. Kimball, Raju V.S. Rajala, Alexander J. Smith, Robin R. Ali, Steven F. Abcouwer, Thomas W. Gardner

**Affiliations:** 1 Ophthalmology & Visual Sciences, University of Michigan Medical School, Ann Arbor, Michigan, USA; 2 Molecular & Integrative Physiology, University of Michigan Medical School, Ann Arbor, Michigan, USA; 3 Internal Medicine (MEND Division), University of Michigan Medical School, Ann Arbor, Michigan, USA; 4 Cell and Developmental Biology, University of Michigan Medical School, Ann Arbor, Michigan, USA; 5 Cellular & Molecular Physiology, Penn State College of Medicine, Hershey, Pennsylvania, USA; 6 Departments of Ophthalmology and Physiology, University of Oklahoma Health Sciences Center, Oklahoma City, Oklahoma, USA; 7 Centre for Gene Therapy and Regenerative Medicine, King's College London, England, United Kingdom

**Keywords:** eye, retina, retinal ganglion cell, mRNA translation, protein synthesis, mechanistic target of rapamycin, mTOR complex, mTORC1, puromycin, gene knockout, AAV2, adeno-associated virus 2, AMPK, AMP-activated protein kinase, BMDM, bone marrow–derived macrophage, cKO, conditional knockout, CMV, cytomegalovirus, 2DG, 2-deoxy-glucose, DMEM, Dulbecco's modified Eagle's medium, 4E-BP1, eukaryotic elongation factor 4 binding protein 1, eEF, eukaryotic elongation factor, GCL, ganglion cell layer, IF, immunofluorescence, IPL, inner plexiform layer, IS, inner segment, ivt, intravitreally, MEF, mouse embryo fibroblast, mGFP, membrane-bound GFP, mTOR, mechanistic target of rapamycin, mTORC2, mTOR complex 2, mTORC1, mTOR complex 1, NIH, National Institutes of Health, NFL, nerve fiber layer, pS6, phosphorylated ribosomal protein S6, Raptor, regulatory-associated protein of mTOR, RBPMS, RNA-binding protein with multiple splicing, RGC, retinal ganglion cell, Rictor, rapamycin-insensitive protein of mTOR, RT, room temperature, SUnSET, SUrface SEnsing of Translation, TBS, Tris-buffered saline, TOP, 5' terminal oligopyrimidine tract

## Abstract

Mechanistic target of rapamycin (mTOR) and mTOR complex 1 (mTORC1), linchpins of the nutrient sensing and protein synthesis pathways, are present at relatively high levels in the ganglion cell layer (GCL) and retinal ganglion cells (RGCs) of rodent and human retinas. However, the role of mTORCs in the control of protein synthesis in RGC is unknown. Here, we applied the SUrface SEnsing of Translation (SUnSET) method of nascent protein labeling to localize and quantify protein synthesis in the retinas of adult mice. We also used intravitreal injection of an adeno-associated virus 2 vector encoding Cre recombinase in the eyes of *mtor-* or *rptor*-floxed mice to conditionally knockout either both mTORCs or only mTORC1, respectively, in cells within the GCL. A novel vector encoding an inactive Cre mutant (CreΔC) served as control. We found that retinal protein synthesis was highest in the GCL, particularly in RGC. Negation of both complexes or only mTORC1 significantly reduced protein synthesis in RGC. In addition, loss of mTORC1 function caused a significant reduction in the pan-RGC marker, RNA-binding protein with multiple splicing, with little decrease of the total number of cells in the RGC layer, even at 25 weeks after adeno-associated virus-Cre injection. These findings reveal that mTORC1 signaling is necessary for maintaining the high rate of protein synthesis in RGCs of adult rodents, but it may not be essential to maintain RGC viability. These findings may also be relevant to understanding the pathophysiology of RGC disorders, including glaucoma, diabetic retinopathy, and optic neuropathies.

The retina is affected by multiple systemic and ocular diseases, yet the mechanisms by which vision is impaired often remains uncertain given the complex changes in retinal blood vessels, neurons, and glial cells of the neurovascular unit (1, 2). The metabolic and structural features of the retina are integrally linked to support phototransduction, signal integration within the retina, and signal transmission to the brain (3, 4, 5). The retina has one of the highest overall metabolic rates of any tissue, greater than brain or myocardium (6). We previously investigated the regulation of retinal protein synthesis and observed: (1) as measured by incorporation of intravenously administered [^3^H]-phenylalanine, retina from normal adult rats exhibited a two-fold higher basal protein synthesis rate than did gastrocnemius muscle, (7); (2) inhibition of glycolysis by 2-deoxy-glucose (2-DG) or 2-fluoro-deoxy-glucose treatment of *ex vivo* retinas reduced retinal protein synthesis, coinciding with phosphatase-dependent dephosphorylation of eukaryotic elongation factor 4 binding protein 1 (4E-BP1) (8); and (3) insulin-deficient diabetic rats exhibited reduced retinal protein synthesis, which was normalized by either subconjunctival delivery of low doses of insulin or by lowering blood glucose levels *via* sodium–glucose-linked glucose transporter 2 inhibition (7). Similarly, Chihara *et al.* (9, 10) employed intravitreal [^3^H]-leucine injection and found reduced retinal incorporation of the label in insulin-deficient diabetic rabbits. However, these studies did not allow the localization of the synthetic defect or its molecular mechanisms to be defined.

Photoreceptors are considered the most metabolically active cells in the retina (11). They are exceptional in that they exhibit a high rate of aerobic glycolysis (the Warburg effect), express high levels of hexokinase 2, and express the tumor-associated pyruvate kinase isozyme M2 in spite of their postmitotic state (12, 13, 14). In addition, photoreceptors contain a relatively high density of mitochondria in their inner segments (ISs) (11). The ISs are thought to maintain high rates of lipid and protein synthesis to support the production of membranous disks containing the phototransduction machinery, which are shed daily in the form of outer segments (12, 14). However, previous studies have suggested that neurons in the inner retina also exhibit appreciable protein synthesis rates. In 1971, Karlsson and Sjöstrand (15) demonstrated remarkably strong labeling of retinal ganglion cell (RGC) after intravitreal [^3^H]-leucine injection in the rabbit. Similar findings were later published by Leon *et al.* (16). Steinman and Ames (17) also noted that RGCs were the site of intense ^3^H-leucine incorporation in rabbit retinas labeled *ex vivo*.

The mechanistic target of rapamycin (mTOR) kinase controls cellular growth, metabolism, proliferation, and survival in response to environmental cues, including growth factor stimuli and availability of nutrients (18). mTOR forms the core for two multiprotein complexes: mTOR complex 1 (mTORC1) containing the protein Raptor (regulatory-associated protein of mTOR), and mTOR complex 2 (mTORC2) containing the protein Rictor (rapamycin-insensitive protein of mTOR). mTORC2 is activated in response to external stimuli, such as insulin, growth factors and cytokines. mTORC2 phosphorylates a limited set of substrates, the AGC kinases, including Akt (Akt serine/threonine kinase), serum, and glucocorticoid-regulated kinase and PKC isoforms (19). mTORC2 is a positive regulator of mTORC1 activity and cell metabolism (20). mTORC1 is also controlled by nutrient availability, cellular energy status, and cell stresses. The two complexes are interconnected, resulting in complex feedback mechanisms that help to reestablish cellular homeostasis under altered environmental conditions (21, 22). Active mTORC1 stimulates metabolic processes, including lipid synthesis, nucleotide synthesis, ribosome production, and protein synthesis, particularly 5′ cap-dependent mRNA translation (19). During brain development, mTORC1 is central to the control of neurogenesis, neural stem cell migration, dendrite formation, and axonogenesis (23, 24). The role of mTOR signaling in mature neurons is less well known, but studies suggest a role for mTORC1 in the control of synaptic plasticity, learning, and memory (25). This dependence is most likely because of the need for tight control of mRNA translation during synapse formation and strengthening.

Relatively little is known about the role of mTOR signaling in retinal physiology and disease. Probing for phosphorylated ribosomal protein S6 (pS6), an indirect but robust assay of mTORC1 activity *in situ*, indicated a large number of cells in the nascent ganglion cell layer (GCL) of the mouse embryo with high mTORC1 activity (26). Hyperactivation of mTORC1 in retinal precursor cells accelerated proliferation and neurogenesis during development, leading to enlarged eyes and increased numbers of retinal neurons (27, 28). Ma *et al.* (29) utilized conditional knockout (cKO) of *mTOR*, *Rptor*, and *Rctor* to examine the role of mTORCs in cone photoreceptor viability and function. Neither lack of mTORC1 (*rptor* cKO) nor lack of mTORC2 (*rctor* cKO) affected long-term cone survival or function, whereas loss of both complexes (*mtor* cKO) slightly diminished cone function without loss of viability. However, loss of mTORC1 accelerated cone death in retinal degeneration models, whereas genetic hyperactivation of mTORC1 in cones by *Tsc1* (tuberous sclerosis complex 1) cKO promoted their survival (30). In contrast to these results in cone photoreceptors, several studies showed that mTORC1 plays a key role in promoting RGC axon regeneration. For example, in the optic nerve crush model of axon regeneration, deletion of phosphatase and tensin homolog promotes RGC axon regeneration, and this effect is dependent upon activation of mTORC1 downstream of Akt (reviewed in Ref. (31)).

We recently showed that the mTOR and other mTORC1 constituent proteins are most highly expressed in RGC in normal humans and rodents (32). Here, we employed the SUrface SEnsing of Translation (SUnSET) method of protein synthesis analysis (33, 34) to determine relative mRNA translation rates in retinal layers and RGCs. The SUnSET method has been applied *in vivo* to measure total protein synthesis rates in several tissues (34, 35), including retina (36, 37), but had not been used to localize protein synthesis in the retina. We used intravitreal injection of an adeno-associated virus 2 (AAV2)-Cre vector to perform conditional knockdown of *mtor* or *rptor*, primarily in the GCL. In addition, we constructed an inactive Cre recombinase–expressing AAV2 vector that provides an optimal control for retinal gene deletion studies. Collectively, the results confirm that RGC exhibits a relatively high rate of protein synthesis that may be important for their function. In addition, we demonstrate that mRNA translation in RGC is sensitive to the glycolysis inhibitor 2-DG and is highly dependent upon mTORC1 function. However, lack of mTORC1 function did not cause a commensurate loss of cells in the GCL.

## Results

### Validation of SUnSET method

**Figure 1:**
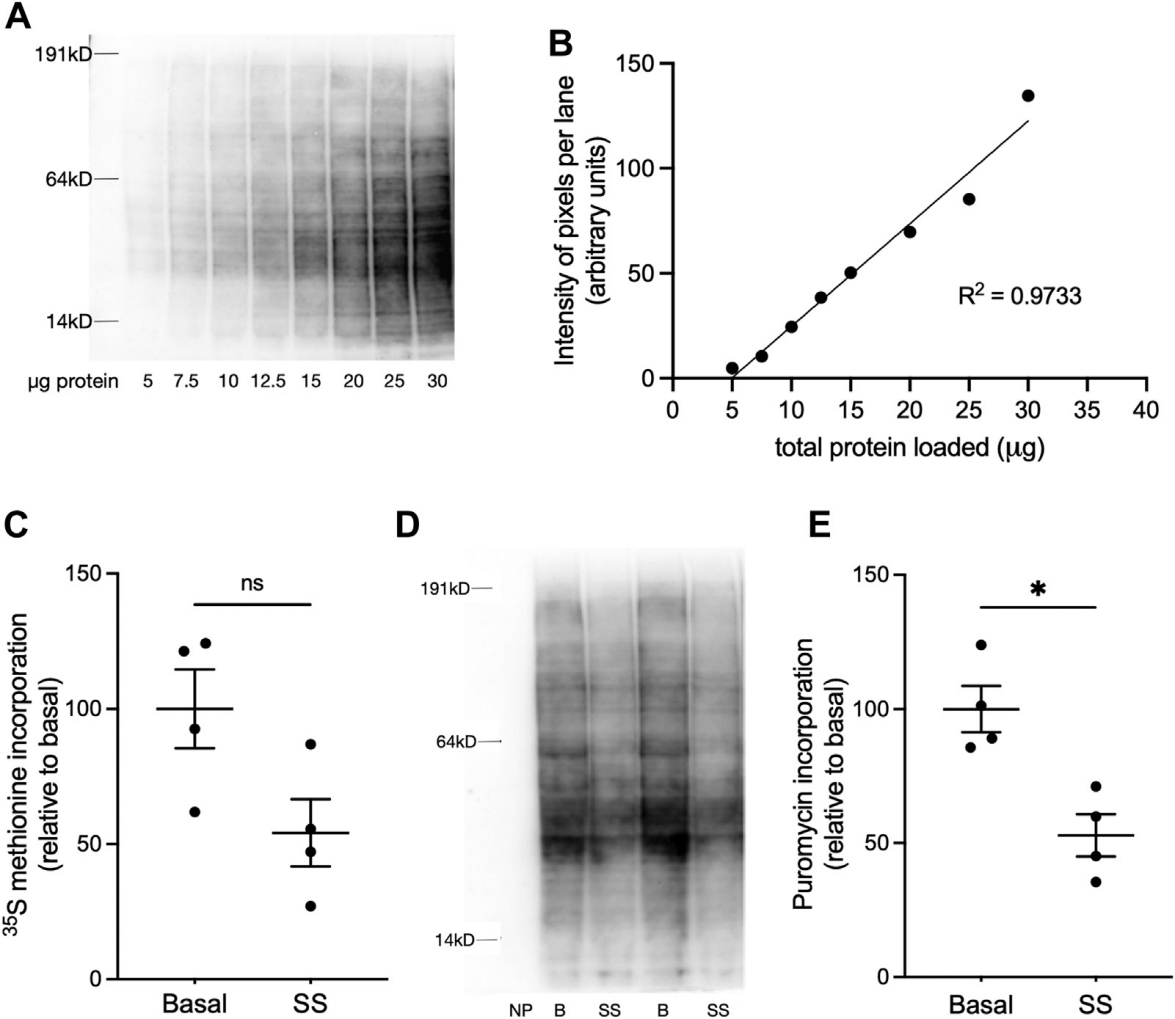
**Comparison of protein synthesis assay methods.***A*, Western blot showing linearity of the R28 lysate samples blotted for puromycinylated protein content. *B*, quantification of Western blot data in *A* showing the correlation between protein loaded per well and total puromycinylated antibody signal integrated over all gel mobilities. (*R*^2^ value = 0.9733). *C*, effect of 4 h serum starvation on R28 cell S^35^-methionine incorporation into precipitable proteins. *D* and *E*, effect of 4 h serum starvation determined by Western blot SUnSET method. NP indicates a control sample not treated with puromycin, B indicates basal (serum fed) samples, and SS indicates 4 h serum-starved samples. Data are shown as mean ± SEM, n = 4/group; ∗*p* ≤ 0.05 by Mann–Whitney *U* test. SUnSET, SUrface SEnsing of Translation.

The SUnSET method of protein synthesis analysis measures incorporation of puromycin, a structural analog of tyrosyl-transfer RNA, into elongating peptide chains, using an antibody that specifically binds puromycinylated peptides (33, 34). To further validate the SUnSET Western blotting method of evaluating protein synthesis, we first compared it with the gold standard method of metabolically labeling nascent protein by ^35^[S]-methionine incorporation using cultured R28 retinal neuron-like cells. As previously noted by Goodman and Hornberger (34), puromycin incorporation occurred in proteins with a wide range of electrophoretic mobilities, as shown in a Western blot and probing with an antibody to puromycinylated proteins (Fig. 1*A*). Quantification of puromycinylated protein content as a function of total cell protein loaded in the gel showed high linearity (*r*^2^ = 0.9733) up to 30 μg/lane (Fig. 1*B*), such that 30 μg/lane loading was used in subsequent assays. Serum starvation was used to manipulate the mRNA translation rate of R28 cells prior to protein synthesis measurements. Both the ^35^[S]-methionine incorporation and SUnSET methods indicated a near 50% decrease in rate of protein synthesis caused by 4 h of serum deprivation (Fig. 1, *C*–*E*). We next assessed puromycinylation of retinal proteins *in vivo* using 100, 400, and 800 mg/kg body weight doses of puromycin given *via* intraperitoneal injection to mice and found the greatest incorporation at 400 mg/kg body weight (Fig. S1). However, mice treated with 400 and 800 mg/kg doses became moribund, so subsequent experiments used a dose of 200 mg/kg body weight, which had no effect on their activity levels. This dose is 9.2-fold higher than the 0.04 μmol/g body weight (21.7 mg/kg) used in previous studies (34, 35). We also examined the use of dot blotting to more easily quantify puromycinylation in retinal tissue lysates by examining the relationship between amount of total protein loaded per dot and the signal obtained (Fig. S2). We found that the signal was relatively linear with loadings of up to 10 μg/well of total protein (approximately 30 μg/mm^2^). The results established methods for SUnSET analysis by Western blotting and dot blotting in both cultured retinal cells and *in vivo* retinas and confirm that the SUnSET method can be used to reliably assess changes in retinal protein synthesis rate.

### Relative rate of protein synthesis in retinal layers

**Figure 2:**
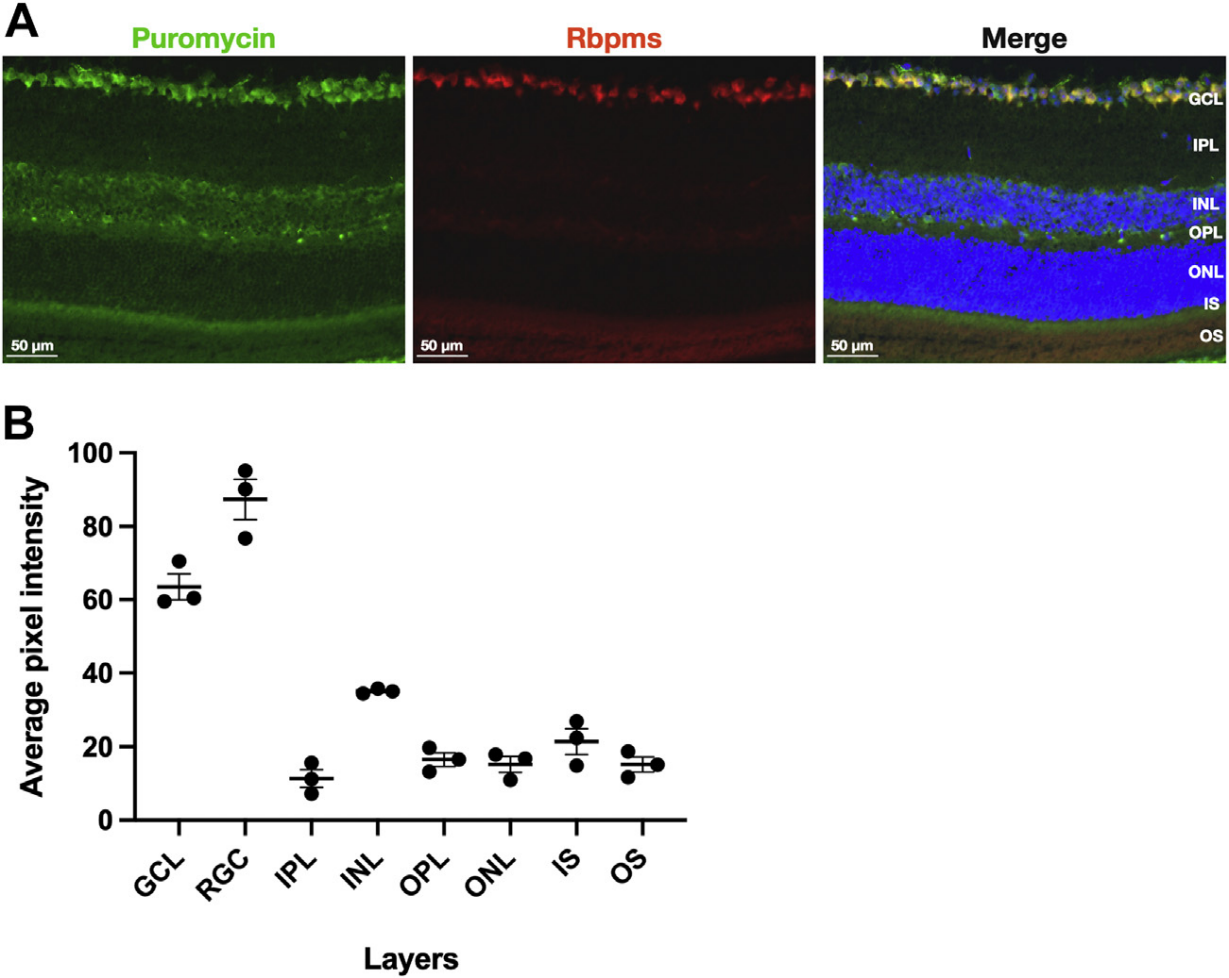
**Relative puromycin incorporation in neural retina layers show highest rates in the GCL and RGC.***A*, representative images of puromycinylation (*green*) and RBPMS IF (pan RGC marker, *red*) and Hoechst staining of nuclei (*blue*) in retinal sections of naive mice. *B*, quantification of puromycin incorporation in each of the retinal layers, shown as average pixel fluorescence intensity. Data are shown as mean ± SEM, n = 3/group. GCL, ganglion cell layer; IF, immunofluorescence; RBPMS, RNA-binding protein with multiple splicing; RGC, retinal ganglion cell.

Using a flooding dose [^3^H]-phenylalanine method, we previously found that the rate of protein synthesis in retinas of normal adult rats and mice was approximately 2-fold greater than that in gastrocnemius muscle (7). However, we did not examine the distribution of protein synthesis in the various retinal layers. The neural retina of mammals, including mouse, is composed of seven distinct layers, including (from inner to outer retina): the nerve fiber layer (NFL) composed of RGC axon bundles, the GCL composed of RGC and displaced amacrine cells, the inner plexiform layer (IPL), the inner nuclear layer, the outer plexiform layer, the outer nuclear layer, and finally the ISs and outer segments of photoreceptors. We used the *in situ* SUnSET method to localize protein synthesis in mouse retinas (Fig. 2). Puromycin incorporation was quantified in each retinal layer (with the NFL included with the GCL) and was found to be highest by far in the GCL, being approximately twice that of the layer with the next highest puromycin incorporation (the inner nuclear layer). Other layers exhibit intensities that are 12.5% to 24.9% of that in the GCL (Fig. 2*B*). The protein synthesis rate was particularly high in RGC soma within the GCL (masked with RNA-binding protein with multiple splicing [RBPMS] immunoreactivity), with a puromycin incorporation density 37% higher than the average of the total GCL.

### Effects of 2-DG treatment on retinal protein synthesis

**Figure 3:**
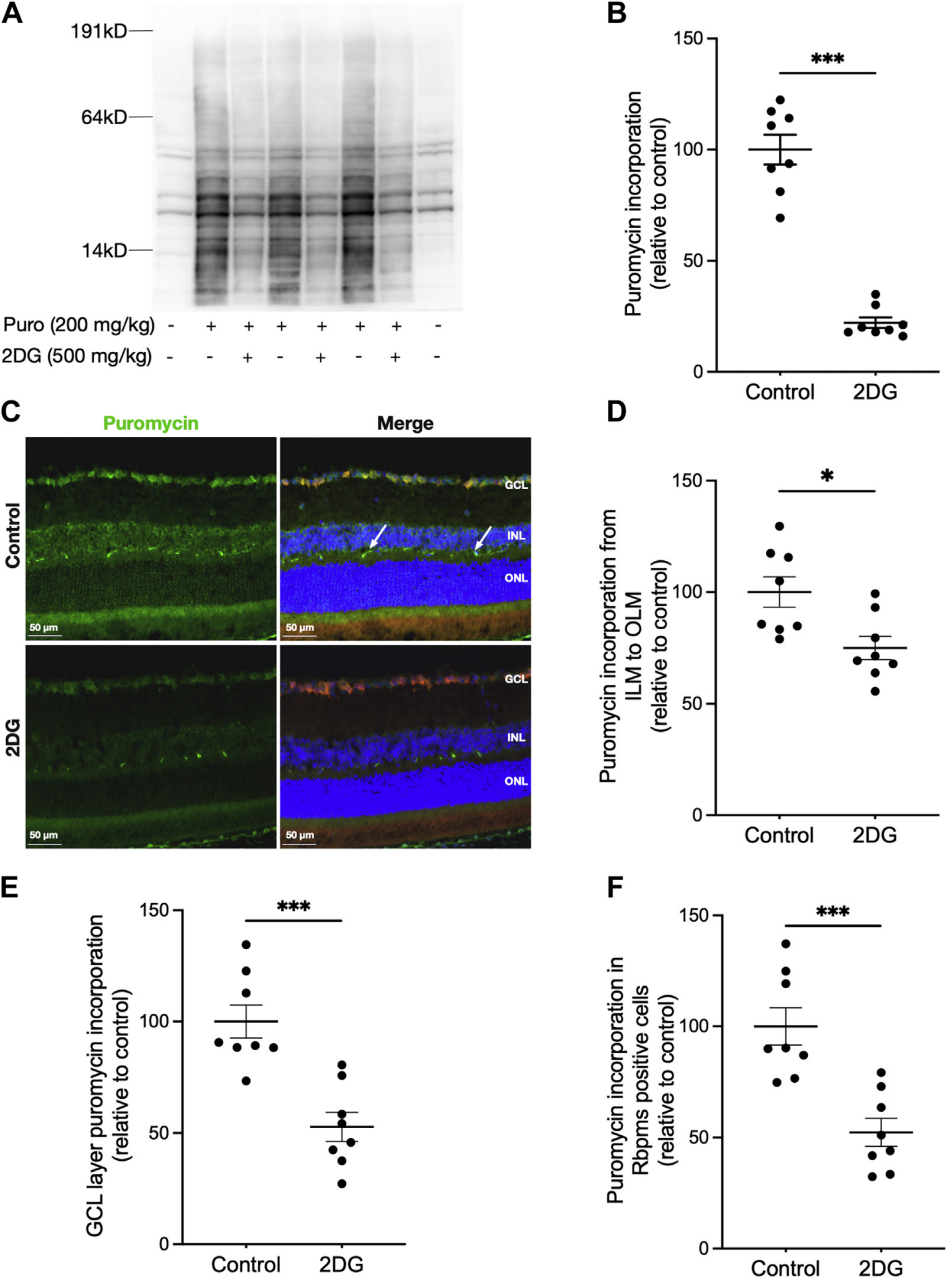
**SUnSET assay shows that 2-DG treatment reduced retinal protein synthesis *in vivo*.***A*, Western blot analysis of protein puromycinylation in retinas of mice with and without 2-DG treatment at 30 min prior to systemic puromycin administration. Control samples from mice that received no puromycin were used as background control. *B*, quantification of SUnSET Western blotting shows the effect of 2-DG treatment on relative puromycin incorporation. Data are shown as mean ± SEM, n = 8/group. ∗∗∗*p* ≤ 0.0001 by Mann–Whitney *U* test. *C*, IF analysis protein puromycinylation (*green*) in retinas of mice with and without 2-DG treatment at 30 min prior to systemic puromycin administration. RBPMS IF (*red*) and Hoechst staining of nuclei (*blue*) are also shown in the merged panel. *Arrows* points to the background staining of blood vessels in the OPL. *C*, quantification of the puromycin incorporation in whole retinal cross sections from the inner limiting membrane (ILM) to the outer limiting membrane (OLM). *E*, quantification of puromycinylation in the GCL only. *F*, quantification of the puromycin incorporation in RBPMS-positive cell somas. Data are shown as mean ± SEM, n = 8/group. ∗∗∗*p* ≤ 0.0001, ∗*p* ≤ 0.05 by Mann–Whitney *U* test was used for statistics. 2-DG, 2-deoxy-glucose; GCL, ganglion cell layer; IF, immunofluorescence; OPL, outer plexiform layer; RBPMS, RNA-binding protein with multiple splicing; SUnSET, SUrface SEnsing of Translation.

We previously demonstrated that *ex vivo* treatment of retinas with 2-DG and 2-fluoro-deoxyglucose rapidly inhibited protein synthesis (8). Therefore, in this study, we used systemic 2-DG treatment of mice to inhibit retinal protein synthesis, which was first evaluated by the Western blot SUnSET method (Fig. 3*A*). This approach indicated a highly significant (*p* < 0.001) 80% reduction in total retinal protein synthesis in response to 2-DG (Fig. 3*B*). The effect appears uniform across the range of protein mobilities. The *in situ* SUnSET method (Fig. 3*C*) indicated that 2-DG treatment significantly (*p* < 0.05) reduced immunofluorescence (IF) by an average of 25% when IF intensity was integrated across all retinal layers (Fig. 3*D*). Thus, the *in situ* SUnSET assay did not quantitatively duplicate the 2-DG effect on whole retina protein synthesis measured with the Western blot SUnSET method; we speculate that this may be due to background IF in the *in situ* method, most notably the blood vessels in the plexiform layers, which are reactive to the antimouse immunoglobulin G secondary antibody and are not affected by 2-DG treatment (*arrows* in Fig. 3*C*). Consistent with that hypothesis, the *in situ* SUnSET method showed that 2-DG treatment significantly reduced puromycin labeling by 50% in both the GCL as a whole (Fig. 3*E*) and in the soma of RGC, as indicated by colocalization with RBPMS (Fig. 3*F*).

The Raptor-containing complex, mTORC1, is a major regulator of protein synthesis in peripheral tissues such as skeletal muscle (35). The Rictor-containing complex, mTORC2, has also been implicated in controlling protein synthesis, but indirectly (38). We recently showed that mTORC1 components are prominently expressed in RGC (32). However, our previous study examining the suppression of retinal protein synthesis by 2-DG indicated that the effect coincided with accelerated dephosphorylation of 4E-BP1 independent of mTORC1 (8). Examination of ribosomal S6 S240/S244 phosphorylation (pS6, a robust indicator of mTORC1 activity) indicated that 2-DG treatment had no effects on retinal mTORC1 activity *in vivo* (Fig. S3). These data show that the SUnSET method can be used to evaluate changes in localized retinal protein synthesis rate and that protein synthesis is relatively high and sensitive to 2-DG treatment in cells within the GCL, including RGC, in an mTORC1-independent manner.

### Effect of mTORC1 cKO on protein synthesis in the GCL

**Figure 4:**
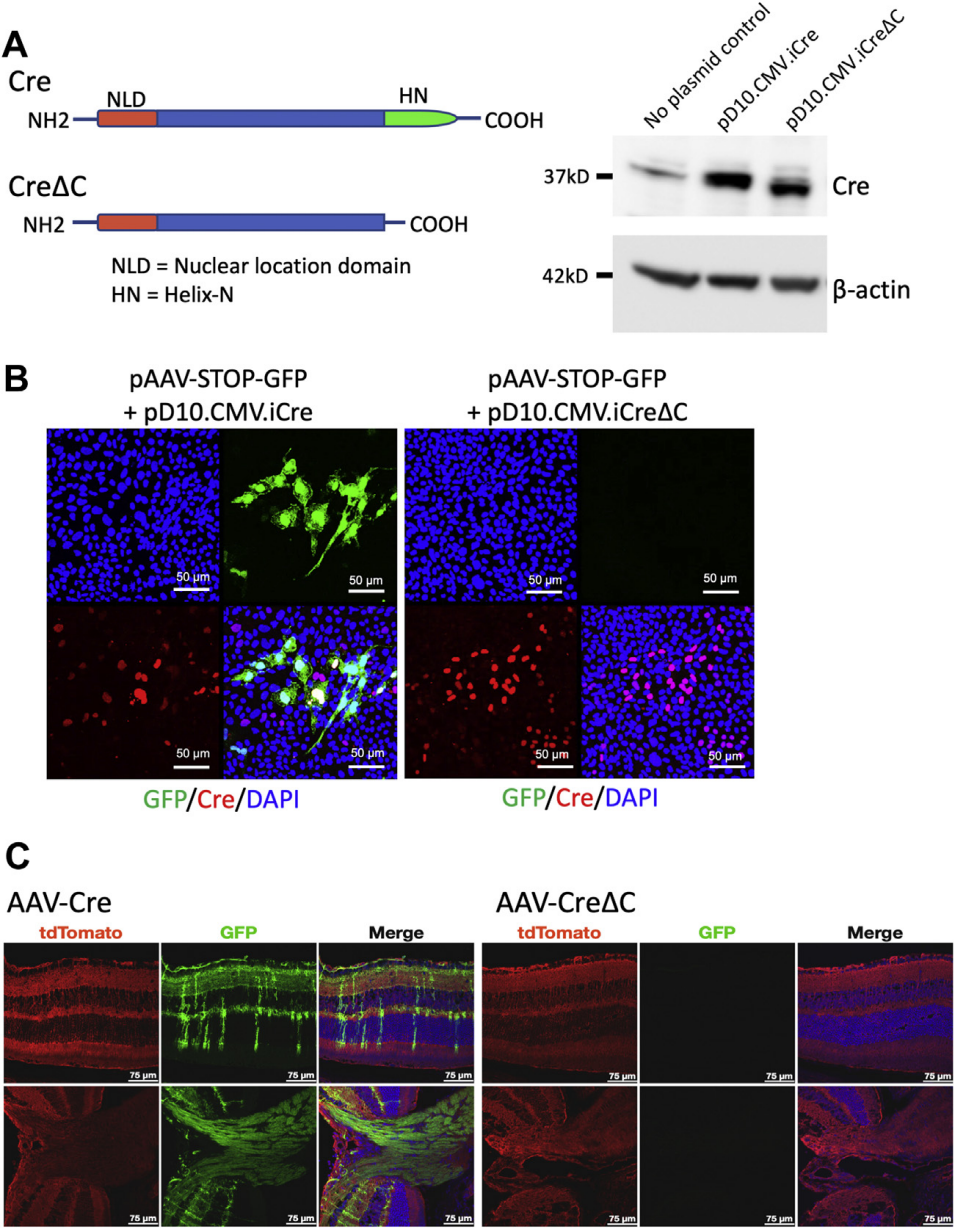
**Construction and testing of a novel control vector expressing a mutant Cre (CreΔC) with no recombinase activity.***A*, diagram showing comparing Cre and CreΔC protein structures indicating the deletion of 12 C-terminal amino acids comprising the helix-N region required for loxP site binding and therefore recombination. A Western blot confirming CreΔC protein expression by probing for Cre and β-actin proteins in BMDMφ transfected with AAV plasmids pD10.CMV.iCre or pD10.CMV.iCreΔC or with no plasmid as control. Note that control lane contains a background band corresponding to an endogenous protein bound by the anti-Cre antibody and that the CreΔC protein exhibits a higher mobility that the wildtype Cre protein. *B*, CreΔC protein recombination was tested by transfecting R28 cells with pD10.CMV.iCre or pD10.CMV.iCreΔC along with pAAV-STOP-GFP reporter plasmid containing a floxed-STOP GFP coding sequence that only expresses GFP after Cre-mediated recombination. Note that the CreΔC plasmid did not enable GFP expression. *C*, *in vitro* testing of recombination at 6 weeks after ivt injection AAV-Cre or AAV-CreΔC viral vectors into the eyes of mT/mG mice. Note that GFP expression indicates recombination in the GCL (note strong GFP expression in the nerve fibers and RGC dendrites localized in the inner IPL), as well as sporadic GFP expression in cells with morphologies indicative of Müller cells. GFP in the nerve bundles at the optic nerve head indicate effective RGC recombination. Note that no GFP was detected in retinas injected with AAV-CreΔC. AAV, adeno-associated virus; BMDM, bone marrow–derived macrophage; GCL, ganglion cell layer; IPL, inner plexiform layer; ivt, intravitreally; RGC, retinal ganglion cell.

We sought to test what factors lead to the relatively high protein synthesis rate in GCL and RGC. The levels of mTORC1 components are relatively high in the mouse GCL and in RGC in particular (32). Although 2-DG apparently did not lower retinal protein synthesis *via* inhibition of mTORC1 activity, we reasoned that it was still possible that an appreciable fraction of protein synthesis in the GCL is dependent upon mTORC1 activity. To examine the necessity of mTORs in support of RGC protein synthesis, we intravitreally (ivt) injected AAV2 viral particles driving expression of Cre under control of a cytomegalovirus (CMV) promoter/enhancer into mice with floxed *mtor* and *rptor* gene alleles. The AAV2 serotype was chosen because of its high tropism for RGC and the resulting high efficiency for gene delivery to RGC after intravitreal injection (39). As a negative control to be ivt injected into the contralateral eye, we constructed an AAV2 encoding a Cre mutant protein containing a 12-amino acid C-terminal deletion (CreΔC) designed to make it incapable of binding to loxP sites (Fig. 4*A*). To characterize this new vector, we first demonstrated that the CreΔC mutant was incapable of causing loxP-mediated recombination by cotransfection of the AAV2 plasmids into R28 cells along with a Cre-reporter vector plasmid containing a Floxed-STOP GFP coding sequence. Whereas the normal Cre caused recombination-induced GFP expression, the CreΔC plasmid showed no activity (Fig. 4*B*). We further tested this vector using primary bone marrow–derived macrophages (BMDMΦ) and mouse embryo fibroblasts (MEFs) derived from mT/mG Cre reporter mice, in which recombination causes a shift from membrane-bound RFP (mtdTomato) to membrane-bound GFP (mGFP) expression. In both MEF cells (Fig. S4*A*) and BMDMφ (Fig. S4*B*), the AAV-Cre plasmid caused activation of mGFP expression, whereas no mGFP expression was caused by the AAV-CreΔC plasmid. Finally, to test the appropriateness of the vectors to cause recombination in the GCL of mice, we ivt injected the AAV2-Cre and AAV-CreΔC into the eyes of mT/mG mice and examined GFP expression 6 weeks after injection (Fig. 4*C*). The Cre vector caused recombination in the vast majority of cells in the GCL as evidenced by the presence of mGFP in the NFL and dendrites within the inner most region of the IPL. Effective recombination in RGC was indicated by mGFP in the majority of axon bundles at the optic nerve head, although some samples suggested more complete recombination in the temporal side of the retina where the injection was placed. Of note, recombination induced by the Cre vector was also detected sporadically in a few cells spanning the whole retina, evidently being Müller cells. In contrast, retinas from eyes injected with the CreΔC vector showed no evidence of mGFP expression.

**Figure 5:**
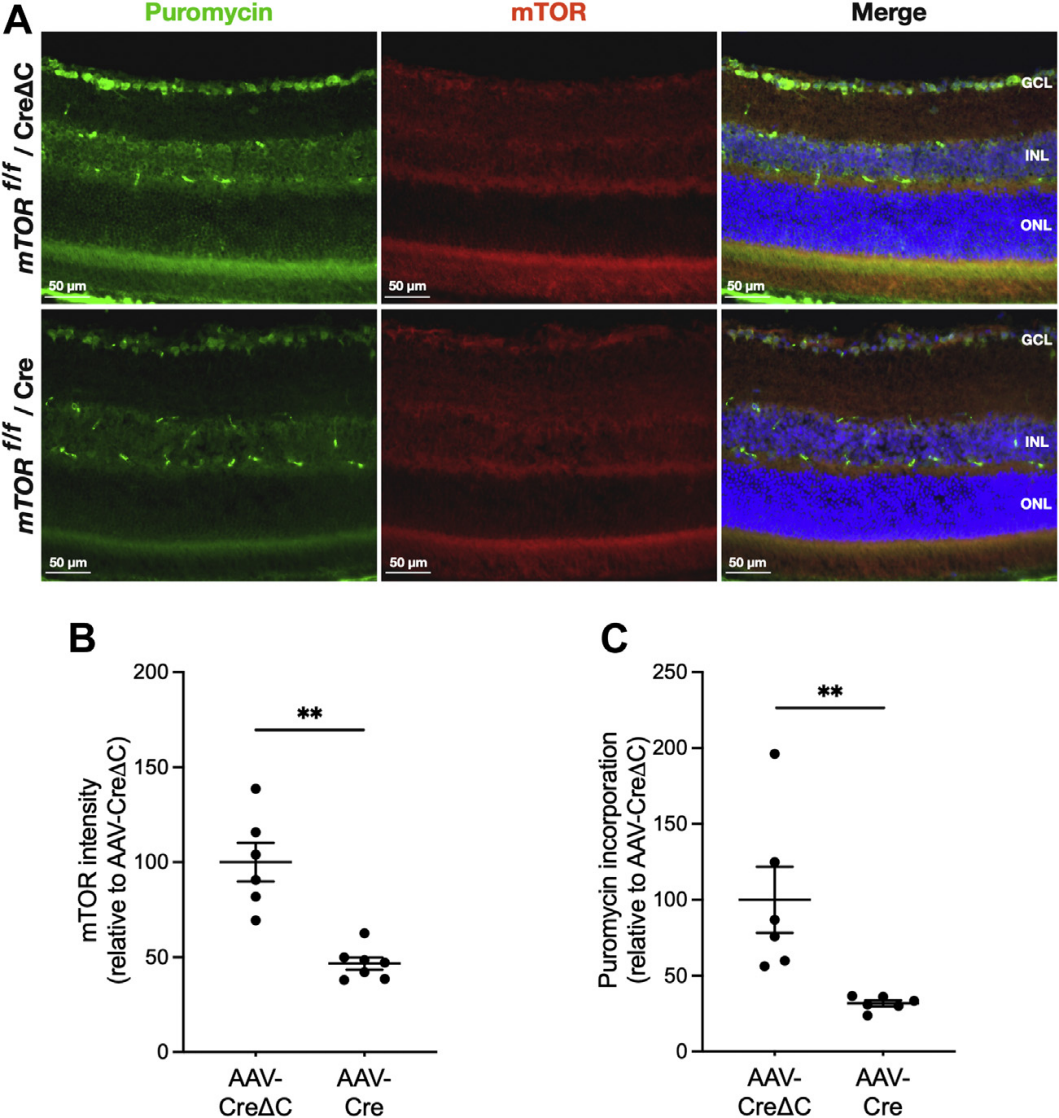
**Conditional knockout of mTOR decreases protein synthesis in the GCL.***A*, representative *in situ* SUnSET assay and mTOR IF probing of retinal sections of *mtor*^*f/f*^ mice 17 weeks after intravitreal injection of AAV-CreΔC (*top rows*) or AAV-Cre (*bottom rows*). Puromycinylated protein IF is shown in *green*, mTOR IF is shown in *red*, and Hoechst staining of nuclei is shown in *blue*. *B*, quantification of mTOR IF intensity in the GCL of retinas at 17 weeks. Intensities were normalized to the mean values of those in the control (AAV-CreΔC) retinas. *C*, quantification of puromycin incorporation in the GCL at 17 weeks. Data are shown as mean ± SEM, n = 6/group. ∗∗*p* ≤ 0.01 by Mann–Whitney *U* test. AAV, adeno-associated virus; GCL, ganglion cell layer; IF, immunofluorescence; mTOR, mechanistic target of rapamycin; SUnSET, SUrface SEnsing of Translation.

**Figure 6:**
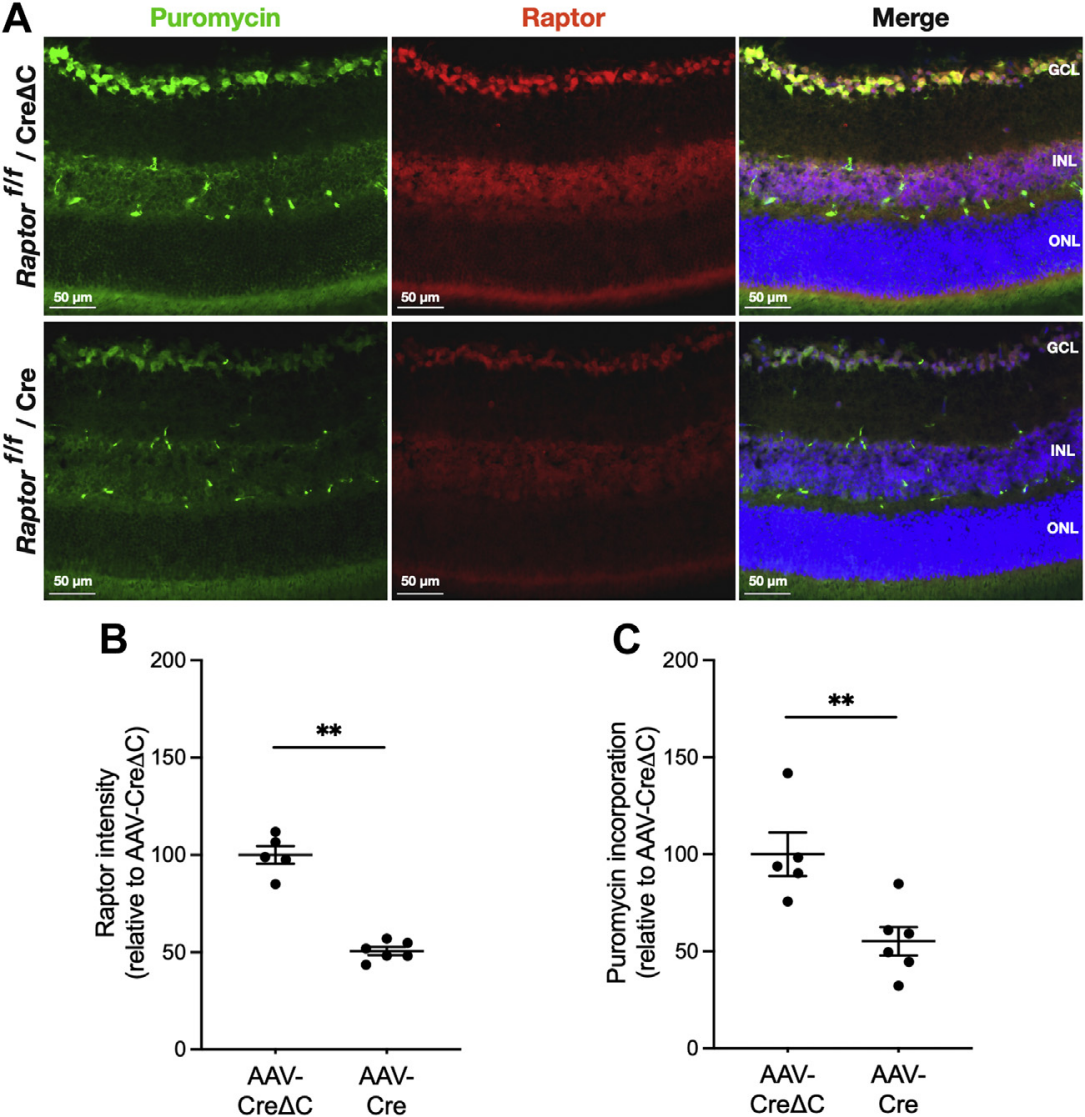
**Conditional knockout of Raptor decreases protein synthesis in the GCL.***A*, representative *in situ* SUnSET assay and Raptor IF probing of retinal sections of *rptor*^*f/f*^ mice at 17 weeks after intravitreal injection of AAV-CreΔC (*top rows*) or AAV-Cre (*bottom rows*). Puromycinylated protein IF is shown in *green*, Raptor IF is shown in *red*, and Hoechst staining of nuclei is shown in *blue*. *B*, quantification of Raptor IF intensity in the GCL of retinas at 17 weeks. Intensities were normalized to the mean values of those in the control (AAV-CreΔC) retinas. *C*, quantification of puromycin incorporation in the GCL at 17 weeks. Data are shown as mean ± SEM, n = 6/group. ∗∗*p* ≤ 0.01 by Mann–Whitney *U* test. AAV, adeno-associated virus; GCL, ganglion cell layer; IF, immunofluorescence; Raptor, regulatory-associated protein of mTOR; SUnSET, SUrface SEnsing of Translation.

To examine the GCL-specific effect of negating mTORC1 and mTORC2 alone or in conjunction, AAV-Cre or AAV-CreΔC vectors were injected ivt into contralateral eyes of *mtor*^*f/f*^, *rptor*^*f/f*^, and *rctor*^*f/f*^ mice. Retinal sections from puromycin-treated mice were then probed with a combination of antibodies to puromycinylated proteins and mTOR, Raptor, or Rictor. Comparing retinas of *mtor*^*f/f*^ mice treated with AAV-Cre and AAV-CreΔC revealed a 51% reduction (*p* < 0.01) of mTOR IF intensity in the GCL (Fig. 5*B*) at 17 weeks after injection. Concurrently, *in situ* SUnSET results showed a 67% reduction (*p* < 0.01) in GCL protein synthesis (Fig. 5*C*). Longer duration postinjection did not increase the effect of mTOR cKO with very similar results after 25 weeks, with 60% reduction of mTOR content (*p* < 0.01) in GCL (Fig. S5, *A* and *B*) and 54% reduction (*p* < 0.01) in GCL protein synthesis (Fig. S5*C*). Specifically targeting mTORC1 by Raptor cKO resulted in a 51% reduction (*p* < 0.01) of Raptor IF in GCL at 17 weeks after injection (Fig. 6, *A* and *B*), which coincided with a 47% reduction (*p* < 0.01) in GCL protein synthesis (Fig. 6*C*). As for mTOR, the effects of Raptor cKO were assessed after 25 weeks and showed 63% reduction (*p* < 0.001) in GCL Raptor IF intensity (Fig. S6, *A* and *B*) and a 76% reduction (*p* < 0.01) in GCL protein synthesis (Fig. S6*C*). The fact that decreases in puromycin incorporation were comparable to the extents of mTOR or Raptor depletion suggests that protein synthesis in the retinal GCL is highly dependent on mTORC1 activity. We also specifically negated mTORC2 in the GCL by ivt AAV2Cre injection into *rctor*^*f/f*^ mice. Application of the same ivt AAV2Cre methods to Rctor-floxed mice had no significant effect on GCL protein synthesis at 17 weeks after injection (Fig. S7*C*), consistent with only mTORC1 being essential for maintaining protein synthesis in the GCL. However, because Rictor IF is high in astrocytes (which are not effectively targeted by AAV2) but is essentially undetectable in other cells of the GCL, we were not able to demonstrate substantial Rictor cKO in the GCL, even 25 weeks after injection (Fig. S7, *D*–*F*).

**Figure 7:**
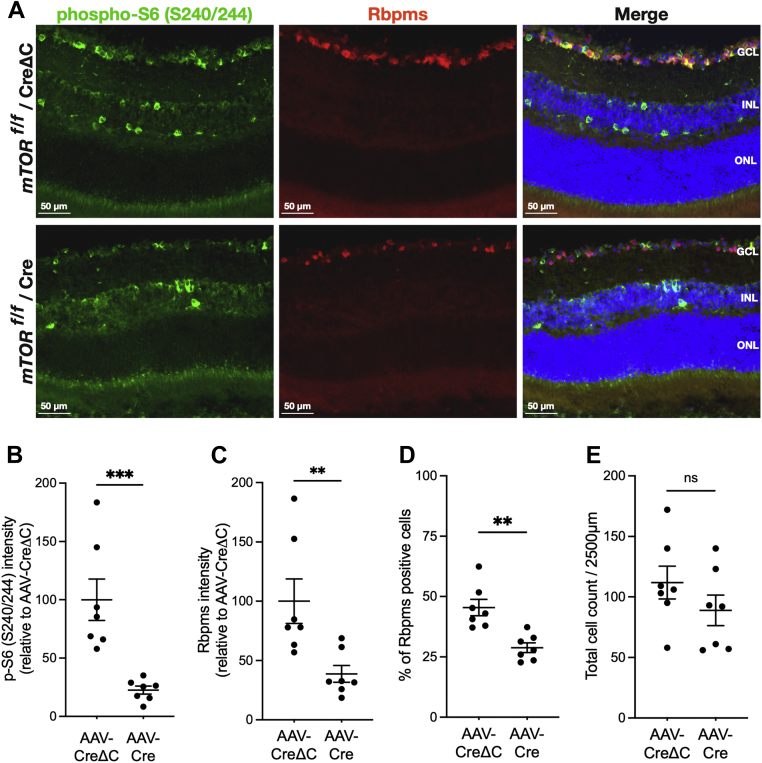
**Conditional knockout of mTOR reduces phosphorylation of ribosomal protein S6 and expression of RBPMS in the GCL.***A*, representative pS6 (S240/S244) and RBPMS IF in retinal sections of *mtor*^*f/f*^ mice at 17 weeks after intravitreal injection of AAV-CreΔC (*top rows*) or AAV-Cre (*bottom rows*). pS6 IF is shown in *green*, RBPMS IF is shown in *red*, and Hoechst staining of nuclei is shown in *blue*. *B*, quantification of pS6 IF intensity in the GCL of retinas at 17 weeks. *C*, quantification of RBPMS IF intensity in the GCL of retinas at 17 weeks. *D*, percentage of RBPMS-positive soma in GCL of retinas at 17 weeks. *E*, total number of cell nuclei per image in the GCL of retinas at 17 weeks. Data are shown as mean ± SEM, n = 7/group. ∗∗*p* ≤ 0.01, ∗∗∗*p* ≤ 0.001 by Mann–Whitney *U* test. AAV, adeno-associated virus; GCL, ganglion cell layer; IF, immunofluorescence; mTOR, mechanistic target of rapamycin; pS6, phosphorylated ribosomal protein S6; RBPMS, RNA-binding protein with multiple splicing.

**Figure 8:**
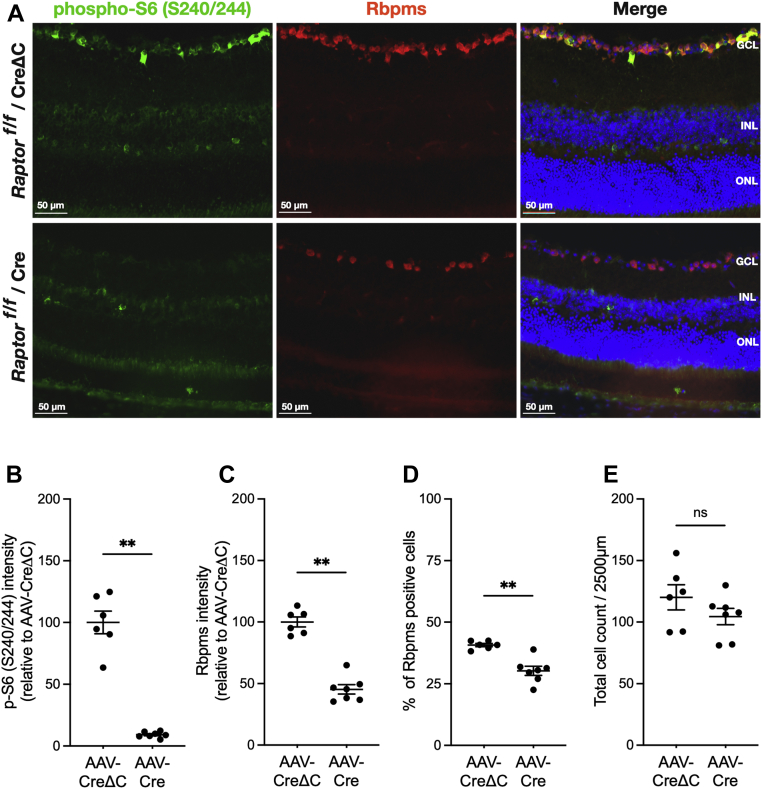
**Conditional knockout of Raptor reduces phosphorylation of ribosomal protein S6 and expression of RBPMS in the GCL.***A*, representative pS6 (S240/S244) and RBPMS IF in retinal sections of *rptor*^*f/f*^ mice at 17 weeks after intravitreal injection of AAV-CreΔC (*top rows*) or AAV-Cre (*bottom rows*). pS6 IF is shown in *green*, RBPMS IF is shown in *red*, and Hoechst staining of nuclei is shown in *blue*. *B*, quantification of pS6 IF intensity in the GCL of retinas at 17 weeks. *C*, quantification of RBPMS IF intensity in the GCL of retinas at 17 weeks. *D*, percentage of RBPMS-positive soma in GCL of retinas at 17 weeks. *E*, total number of cell nuclei per image in the GCL of retinas at 17 weeks. Data are shown as mean ± SEM, n = 6/group. ∗∗*p* ≤ 0.01, ∗∗∗*p* ≤ 0.001 by Mann–Whitney *U* test. AAV, adeno-associated virus; GCL, ganglion cell layer; IF, immunofluorescence; pS6, phosphorylated ribosomal protein S6; Raptor, regulatory-associated protein of mTOR; RBPMS, RNA-binding protein with multiple splicing.

Although no obvious decreases in GCL nuclei density were noted in the previous analysis, we sought to determine if mTOR or Raptor cKO caused loss of RGCs that could affect the protein synthesis results. Retinal sections were probed with an antibody to pS6 (S240/S244) to document diminished mTORC1 activity, along with an antibody to RBPMS to determine RGC density. At 17 weeks after injection of AAV-Cre, mTOR cKO causes an 80% reduction (*p* < 0.001) of total integrated pS6 IF intensity in the GCL (Fig. 7*B*) and a 65% reduction (*p* < 0.01) in pS6 at 25 weeks after injection (Fig. S8*B*), thus confirming diminished mTORC1 activity. This result coincided with significant reductions in total RBPMS IF intensities of 60% (*p* < 0.01) at 17 weeks (Fig. 7*C*) and a 47% (*p* < 0.01) at 25 weeks (Fig. S8*C*). However, it was apparent that this decreased integrated intensity was not simply because of a lower density of RBPMS-positive cells, but rather a reduction of RBPMS expression, as the percentages of RBPMS-positive RGC in the GCL only decreased by about 30% at 17 weeks (Fig. 7*D*) and 25 weeks (Fig. S8*D*). Consistent with this observation, counting all Hoechst-stained nuclei in the GCL confirmed a moderate 22% cell loss at 17 weeks (Fig. 7*E*) and 17% at 25 weeks (Fig. S8*E*). Qualitatively similar results were observed in retinas with Raptor cKO at 17 weeks (Fig. 8) and 25 weeks (Fig. S9) after AAV-Cre injection. The results suggest that a lack of mTORC1 appreciably downregulated RBPMS expression and that the majority of diminished GCL protein synthesis caused by negating mTORC1 activity cannot be attributed to cell death. Thus, most cells in the GCL can survive for an appreciable period without the benefits of mTORC1 activity. These findings also point to the ability to specifically study the mechanisms of mTORC1-regulated RGC protein synthesis without the complication of acute cell death.

## Discussion

In this study, we refined the *in situ* SUnSET method to quantify and localize protein synthesis in the mouse retina and found that the highest levels occur in the GCL and RGC. This observation that RGC and displaced amacrine cells have a high rate of protein synthesis, even relative to photoreceptors, is an essential new insight into the function of the inner retina. This finding is of particular importance given the prominent dysfunction and loss of RGCs in glaucoma, diabetes, as well as hereditary, toxic, and ischemic optic neuropathies. The present findings confirm and extend several early studies that used metabolic labeling with radiolabeled amino acids and autoradiography to examine retinal protein synthesis and documented considerable incorporation into cells in the GCL (16, 40, 41, 42, 43, 44). However, these studies employed *in vitro* metabolic labeling of *ex vivo* retinas. For example, using tritiated phenylalanine incorporation into *ex vivo* rabbit retinas, Maraini and Franguelli (40) reported intense labeling of RGC. However, because of the sparseness of cells in the GCL, counts of radioautographic silver grains formed over cells in the GCL were not normalized in a way that could be directly compared with other retinal layers.

Two previous studies applied the SUnSET method to retinas. Starr *et al.* (36, 37) employed the Western blot SUnSET method to investigate the effects of inherited photoreceptor (rd16) degeneration on protein synthesis and found attenuation of mRNA translation in degenerated mouse retinas. However, they did not optimize the puromycin dose or localize incorporation of the label in the retina. The SUnSET method has the advantages that it does not require an exogenous radioactive label, and it permits semiquantitative localization of protein synthesis. A limitation of the SUnSET method is that it does not allow calculation of absolute rates of protein synthesis normalized to protein content, as does the flooding dose [^3^H]-phenylalanine method.

Using the *in situ* SUnSET method, we found that protein synthesis in the GCL and specifically in RGC was greatly inhibited by 2-DG treatment and highly dependent upon mTORC1 signaling. However, the effect of 2-DG treatment did not coincide with a reduction of ribosomal S6 S240/S244 phosphorylation. mTORC1 regulates protein synthesis in skeletal muscle and liver in response to insulin, amino acids, and exercise (45, 46). We previously found that human, rat, and mouse RGC express relatively high levels of mTORC1 constituent proteins (32). 2-DG was tested because we previously found that it effectively inhibited protein synthesis in *ex vivo* retinas (8). A previous study attributed the ability of 2-DG to inhibit neuronal protein synthesis to activation of AMP-activated protein kinase (AMPK) and subsequent phosphorylation of eukaryotic elongation factor 2 (eEF2) (47). Although best known as an inhibitor of glycolysis, 2-DG also inhibits N-linked protein glycosylation, with the toxicity of 2-DG to tumor cells being ascribed to the latter (48). Tailler *et al.* (49) attributed the ability of 2-DG to inhibit mRNA translation to both blocking glycolysis, with subsequent loss of ATP levels, AMPK activation, and mTORC1 inhibition, and inhibition of N-linked glycosylation, with subsequent endoplasmic reticulum stress. That study showed that 2-DG activated the integrated stress response causing deactivation of eukaryotic initiation factor 2-alpha. However, in our previous analysis using *ex vivo* retinas, 2-DG treatment did not increase AMPK, eEF2, or eukaryotic initiation factor 2-alpha phosphorylation (8). Instead, 2-DG caused rapid dephosphorylation of the mTORC1 substrate 4E-BP1, while only lowering total mTOR kinase activity to a relatively small extent. We attributed the rapid dephosphorylation of 4E-BP1 to phosphatase action because the response was largely prevented by inhibition of protein phosphatase 1 and protein phosphatase 2A with okadaic acid and calyculin A, as well as by inhibition of PPM1 with cadmium. The present results suggest that 2-DG treatment also inhibits *in vivo* retinal protein synthesis in an mTORC1-independent manner.

Although mTORC1 is often thought to stimulate general mRNA translation by phosphorylation of ribosomal S6 kinase and 4E-BPs, mTORC1 acutely supports the synthesis of a specific set of proteins. Recently, Umegaki *et al.* (50) used ribosome profiling to determine how the translatome of cultured cortical neurons was affected by acute pharmacological inhibition of mTOR activity. That article confirmed a prior study in non-neuronal cells showing that translation of mRNAs containing 5' terminal oligopyrimidine tract (TOP) or TOP-like motifs is highly dependent on mTORC1 (51). Many of these TOP mRNAs encode proteins making up the translational machinery, including ribosomal proteins, translation initiation factors (eIFs), translation elongation factors (eEFs), and poly-A–binding factors (52). Thus, mTORC1 activation increases the cell's ability to perform general mRNA translation, and loss of mTORC1 function eventually results in inhibition of general mRNA translation by downregulating the cell's protein synthetic capacity. It is likely that this occurs in RGCs lacking mTORC1 because of disruption of *mtor* or *rptor* genes. Future studies investigating the temporal effects of mTORC1 loss on translation of specific proteins are needed to test this hypothesis.

Here, we confirm that mTORC1 is essential for maintaining a relatively high steady-state level of protein synthesis in RGC of healthy adult rodent retina. In the brain, mTORC1 is vital to the proliferation of neuronal precursor cells during development and supports neuronal plasticity and dendrite growth in adults (25). Likewise, mTORC1 supports retinal progenitor cell growth, although lack of mTORC1 results in the overproduction of RGCs in the postnatal retina (53). During development, extensive protein synthesis is needed for RGCs to extend their long axons, a substantial portion of the protein synthesis localizing directly in growth cones (54). After optic nerve crush, enhanced mTORC1 signaling is necessary for the stimulatory effect of phosphatase and tensin homolog deletion on RGC axon regeneration (26, 31). However, the reason that fully grown uninjured adult RGC exhibits a relatively high rate of mTORC1-dependent protein synthesis remains unclear. Our analysis included only the GCL-containing RGC soma. Protein synthesis occurs locally in both RGC soma and axons (55). Using an axon-trap-RiboTag approach, Shigeoka *et al.* (56) compared the translatome of distal RGC axons within the superior colliculus to that of the RGC soma, nerve fibers, and dendrites within the retina. In the adult mouse, the translatome of the distal RGC axons was enriched in genes associated with synaptic transmission as well as cellular metabolism and mitochondrial respiratory chain. It has been well established that a fraction of proteins synthesized in RGC soma are transported into their axons, the optic nerve, and nerve terminals (57). In fact, “transportomes” have been identified that are comprised of proteins produced in RGC soma and transported into the optic nerve, lateral geniculate nucleus, and the superior colliculus (58). However, to the best of our knowledge, the proportion of protein synthesis demand on RGC represented by proteins produced in the soma and transported into axons has not been determined.

Although lack of mTOR or Raptor expression caused a proportional decrease in protein synthesis, it did not cause a dramatic loss of RGC cells, even 25 weeks after AAV-Cre injection. This result suggests that RGC viability is not highly dependent on mTORC1 or mTORC2 activity or on maintenance of a high level of mRNA translation under normal physiological conditions. Similarly, Punzo *et al.* (29, 59) found that cKO of Raptor in cone photoreceptors did not cause the death of those cells. However, these authors did find that loss of mTORC1 accelerates cone cell death after sodium iodate–induced retinal pigment epithelial cell atrophy. We did see a significant decrease in the number of RBPMS^+^ cells in the GCL of *mTOR* and *Rptor* cKO mice. RBPMS is a pan marker for RGC soma in adult rodents (60, 61, 62) and is often used to quantify RGC numbers in neurodegenerative models (63, 64, 65). However, in the present case, the apparent loss of RBPMS-positive cells was largely because of RBPMS content decreasing below the IF threshold and less so to RGC death. Our data suggest that expression of RBPMS is dependent upon mTORC1; however, whether it is directly because of diminished mRNA translation remains to be demonstrated. RBPMS levels are very high in the soma of adult RGC, but its role in RGC function is not well determined. RBPMS is part of a family of RNA-binding proteins that affect RNA stability and translational regulation (62). There is evidence that RBPMS may facilitate the localization of mRNAs to cytoplasmic granules (66). During development of *Xenopus laevis* and zebrafish retinas, RBPMS is found in RNA granules that transport along growing axons and is necessary for axon sorting, synapse, and arbor formation (67, 68). Recently, Pereiro *et al.* (69) used adult retinal explants to demonstrate that RBPMS migrated from RGC soma to the dendrites in the IPL under hypoxic conditions and eventually into degenerative axons. Future studies will examine how lack of mTORC1 affects RGC function, including RBPMS expression and function, as well as its role in RGC survival in stressed conditions.

High metabolic activity of retinal ganglion cells has been suggested for decades; however, its mechanisms of regulation have been widely unknown, in part because of the technical difficulties to quantitatively measure cell-specific metabolism. The present study used the relatively novel SUnSET methodologies combined with AAV-driven genetic manipulations to provide the first clear evidence of the high rate of mRNA translation in RGC and the central role of mTORC1 in the regulation of this protein synthesis. Such a high metabolic demand may provide insight into the high sensitivity of RGCs to diseases such as diabetes and glaucoma.

## Experimental procedures

### Animals

All experiments were conducted in accordance with the National Institutes of Health and Association for Research in Vision and Ophthalmology Statement for the use of Animals in Ophthalmic and Visual research and were approved by the University of Michigan Institutional Animal Care & Use Committee. Male C57BL/6J mice (Jackson Laboratory) were housed under a 12:12 h light–dark cycle with free access to standard chow and water. mT/mG Cre reporter mice (70) were obtained from Jackson Laboratory (stock no.: 007676). *mTOR*^*f/f*^ mice (71) were provided by George Thomas and Sara Kozma (University of Cincinnati). *Rptor*^*f/f*^ and *Rctor*^*f/f*^ mice (72) were provided by Michael Hall (University of Basel). These mice were crossed to the B6 background and routinely tested for the *rd8* mutation (30). For protein synthesis assays, retinal tissue isolations were consistently performed between 10:00 and 11:00 AM to minimize potential circadian variations in protein synthesis as described previously (32).

### Comparison of ^35^S-labeling method with puromycin method of protein synthesis

The R28 retinal precursor cell line (73) was a generous gift from Dr Gail M. Seigel (University at Buffalo). R28 cells were cultured in Dulbecco's modified Eagle's medium (DMEM) plus 10% fetal bovine serum and penicillin–streptomycin antibiotics. For experiments, cultures at 40 to 50% confluence were fed with fresh media with or without fetal bovine serum (serum-starved) 4 h prior to metabolic labeling. For nascent protein labeling with ^35^S-methionine, R28 cells were incubated in DMEM lacking l-methionine for 30 min prior to incubation in DMEM containing 1 mCi/mmol l-[^35^S]-methionine for 60 min. Labeled cells were rinsed with PBS and immediately harvested in lysis buffer (20 mM Hepes, 2 mM EGTA, 50 mM NaF, 100 mM KCl, 0.2 mM EDTA, 50 mM sodium β-glycerophosphate, 1 mM DTT, 1 mM benzamidine, 0.5 mM sodium orthovanadate, 2.5% Triton X-100, 0.25% deoxycholate, plus protease inhibitor) and frozen. To determine ^35^S-methionine incorporation, samples were thawed, centrifuged at 3000*g* for 3 min to clear, and supernatants were transferred to a tube containing five volumes of 1 N trichloroacetic acid, incubated at 100 °C for 15 min, cooled on ice, and then centrifuged at 3200*g* for 10 min. The protein pellets were washed twice with 0.5 N trichloroacetic acid, once with chloroform:ethanol:ether (1:2:1 by volume) and once with diethyl ether, and then dried in room air. The pellets were dissolved in 0.1 N NaOH, and aliquots were scintillation counted in duplicate. Protein concentrations were measured using the Bradford Protein Assay (Bio-Rad). Protein synthesis rate was calculated as CPM per microgram protein per hour and normalized to the basal (serum fed) state to obtain relative values.

For analysis of protein synthesis by the SUnSET Western blot method, R28 cell cultures were incubated in 1 μM puromycin for 30 min. Cells were then rinsed with PBS and subsequently harvested in freshly prepared lysis buffer (50 mM Hepes, 137.5 mM NaCl, 2 mM sodium orthovanadate, 10 mM sodium pyrophosphate, 10 mM NaF, 2 mM EDTA, 2 mM PMSF, 01566% benzamidine, 10% glycerol, 1% NP-40, and protease inhibitor) and centrifuged at 10,000*g* at for 5 min to clear. Supernatant protein concentrations were assessed using the DC Protein Assay (Bio-Rad). Equal amounts (30 μg) of protein were subjected to Western blotting as described previously (7, 32). Blots were blocked and then probed with a mouse monoclonal antibody to puromycinylated protein (Kerafast; catalog no.: EQ0001; 1:1000 dilution) or a rabbit monoclonal antibody to GAPDH (Cell Signaling; catalog no.: 2119; 1:5000 dilution) in Tris-buffered saline (TBS) with 0.1% Tween-20 with 5% nonfat milk. After incubation with secondary antibodies, chemiluminescence (puromycinylated proteins) was quantified using ImageQuant TL (GE Lifesciences), whereas Cy5 fluorescence (GAPDH) was quantified using a Typhoon imager (GE Healthcare). Puromycinylation signals for all protein bands in each lane were integrated and summed. Background values obtained using retinal samples from non–puromycin-treated mice were subtracted. Values were normalized to GAPDH content and then to the mean for the basal condition (serum fed) to obtain relative values.

### *In vivo* protein synthesis assays

For the *in vivo* SUnSET assay, a 25 mg/ml solution of puromycin dihydrochloride (Sigma) was prepared in sterile 0.9% NaCl and i.p. injected at a dosage of 200 mg/kg body weight; the animals were sacrificed, and the retinas were harvested 30 min later. A preliminary time-course experiment determined that the 30 min circulation time was within the linear increase range. Initial experiments examined retinal protein labeling following various doses of puromycin (100, 200, 400, and 800 mg/kg i.p.) and determined that the 200 mg/kg dose was optimal because it was within the range of linear increase in protein labeling and showed no appreciable effect on animal behavior (data not shown). To examine the effect of inhibiting glycolysis on retinal protein synthesis, 500 mg/kg of 2-DG (100 mg/ml in 0.9% NaCl) was injected i.p. and allowed to circulate for 30 min before puromycin injection. This dose of 2-DG was obtained from a previous study (74).

For analysis of protein synthesis rate by the SUnSET Western blot method, retinas were lysed and protein immunoblotted as described for R28 cells (aforementioned). Dot blots were also performed using nitrocellulose membranes in a Minifold-I-Dot-Blot 96-well apparatus (Schleicher and Schuell) and loading 5 μg total protein per lane, unless otherwise indicated. Antibody incubations were the same as those described for R28 cells (aforementioned). Background signal, principally derived from binding of the antimouse immunoglobulin G secondary antibody binding to endogenous immunoglobulins in retinal lysates, was derived from retinal samples from mice not treated with puromycin and subtracted.

For *in situ* localization of retinal nascent protein synthesis by the SUnSET method, mice were treated with puromycin and then whole eyes were enucleated and fixed in 4% paraformaldehyde for 1 h at room temperature (RT) and rinsed in PBS. The corneas and lenses were removed, and the eye cups were processed through sucrose gradient incubations and then mounted in optimum cutting temperature compound (Sakura Finetek) and frozen on dry ice. Frozen sections (10 μm thickness) were rehydrated in PBS, permeabilized for 15 min in PBS with 0.3% Triton X-100, incubated in blocking solution (3% donkey serum and 1% bovine serum albumin in PBS) for 30 min at RT before incubating overnight at 4 °C in the following primary antibodies and dilutions: mouse antipuromycinylated proteins (Kerafast; 1:750 dilution), rabbit monoclonal anti–phospho-S6 ribosomal protein (S240/S244; Cell Signaling Technologies; catalog no.: 5364; 1:800 dilution), guinea pig polyclonal anti-RBPMS (Millipore; catalog no.: ABN1376; 1:400 dilution), rabbit polyclonal anti-mTOR (Cell Signaling Technologies; catalog no.: 2972; 1:50 dilution), rabbit polyclonal anti-Raptor (Millipore; catalog no.: 09-217; 1:50 dilution), goat polyclonal anti-tdTomato (LSBio; catalog no.: LS-C340696; 1:800 dilution), and rabbit polyclonal anti-GFP (Invitrogen; catalog no.: A11122; 1:500 dilution). We previously validated the mTOR, Raptor, S6, RBPMS, and Raptor antibodies for immunohistochemistry (32). Sections were incubated with the appropriate secondary antibodies conjugated to Alexa Fluor dyes, AF488 or AF594 (Life Technologies). Nuclei were counterstained with Hoechst 33342 (Life Technologies). Negative controls were performed by omitting the primary antibodies. IF images were acquired with a Leica DM6000 microscope using a Leica DFC365FX camera (Leica Biosystems). Images were captured using the Leica Application Suite (LAS, version 3.4.2.18368) using consistent light intensities and exposures as described (7). Images obtained from four to eight sections per animal were analyzed.

For quantitative analysis of SUnSET images, all parameters (antibody concentrations and acquisition parameters) were kept identical to limit sources of variability. Images were analyzed in a blind fashion using masking in Fiji (version 2.0.0-rc-69/1.52p, https://imagej.net/software/fiji/), a distribution of ImageJ (75). To restrict the analysis to the soma of the cells in the GCL layer and ignore background outside the cells, images were analyzed through a dual approach: the region of interest on each image was first restricted to the GCL based on histology and staining for RBPMS. Images were duplicated, and a copy was subjected to background subtraction (rolling ball method), and then intensity threshold was adjusted to create a mask covering all the cell soma. This mask was subsequently used to restrict the quantification of the integrated density (sum of all signals) to positive cells; on the other, unaltered image by using the particle analysis feature and ignoring individual clusters of fewer than five pixels. The result was expressed as a sum of the integrated densities for the entire image. An average integrated density of puromycinylated protein per cell was obtained by dividing the integrated density quantified by the number of nuclei in the GCL. The image of Hoechst staining was used to count nuclei using Fiji, as previously described (76). Briefly, after contrast enhancement using the Enhance Local Contrast (CLAHE; http://imagej.net/Enhance_Local_ Contrast_(CLAHE) (77), individual nuclei were automatically counted using the Image-Based Tool for Counting Nuclei plug-in (ITCN, version 1.6; Center for Bio-Image Informatics; http://www.bioimage.ucsb.edu/automatic-nuclei-counter- plug-in-for-imagej). For quantifcation of puromycinylation in the individual layers of the retina, using Fiji retinal layers were manually selected based on histology. Background intensity was subtracted using a rolling ball method, and intensity-based thresholding was performed separately for each layer.

### Development and production of AAV-Cre vectors

A negative control mutant Cre-recombinase (CreΔC) was generated by deleting the C-terminal 12 amino acids of codon-improved Cre (iCre (78)), thus negating its ability to bind loxP sites (79). A 340 bp DNA fragment encoding the truncated C terminus of iCre was synthesized and substituted for the 376 bp NotI–BstEII fragment of pD10.CMV.iCre AAV plasmid to create pD10.CMVCreΔC. To test the negating effect of the C-terminal deletion, the wildtype and mutant AAV plasmids were transfected into R28 cells, as well as BMDMΦ and MEF derived from mT/mG Cre reporter mice. Subconfluent R28 cells were cotransfected with pD10.CMV.iCre or pD10.CMV.iCreΔC together with pAAV-stop-GFP plasmid (80) at a 1:1 mass ratio using Lipofectamine 3000 (Invitrogen). BMDMΦ cells were derived from adult mT/mG mice using an established protocol (81) with the following modifications: the bone-marrow cells were cultured in DMEM supplemented with 30% L929 cell conditioned medium to stimulate growth and differentiation into macrophages. The bone marrow cells were plated and cultured on untreated polystyrene petri dishes, and nonadherent cells were discarded. mT/mG BMDMΦ were electroporated with pD10.CMV.iCre or pD10.CMV.iCreΔC using a Neon Transfection System (Thermo Fisher Scientific), using buffer R and a setting of 1500 V, 20 mS, and 1 pulse. MEFs were derived from E18.5 mT/mG embryos using an established protocol (82), except that the cells were cultured on tissue culture–treated flasks and plates treated with 2% gelatin. mT/mG MEFs were transfected with pD10.CMV.iCre or pD10.CMV.iCreΔC plasmids using TransIT-2020 transfection reagent (Mirus Bio) following the manufacturer's protocol. For each cell type, expression of Cre protein, tdTomato, and/or GFP was examined 3 days after transfection. For Western blots of Cre expression, 25 μg of protein lysates were loaded and membranes were probed with rabbit polyclonal anti-Cre (Millipore; catalog no.: 69050-3; 1:2000 dilution) followed by mouse monoclonal anti-β-actin (Sigma; catalog no.: A5316; 1:2000 dilution). For IF analysis, cells were washed with PBS three times, permeabilized with 0.5% Triton X-100 in TBS for 15 min at RT, blocked with 10% donkey serum plus 0.5% Triton X-100 in TBS for 1 h at RT, and then probed with rabbit polyclonal anti-Cre (Millipore; 69050-3; 1:75 dilution), goat polyclonal anti-tdTomato (LSBio; catalog no.: LS-C340696; 1:500 dilution), and rabbit anti-GFP (Invitrogen; catalog no.: A21311; 1:200 dilution) in blocking buffer for 1 h at RT.

Wildtype and negative mutant Cre recombinase–encoding AAV2.2 vectors (AAV2Cre and AAV2CreΔC) were generated by bipartite transfection of pD10.CMV.iCre or pD10.CMVΔCre plasmids, respectively, with helper plasmids encoding AAV serotype 2 Rep and Cap proteins and adenovirus helper functions into human embryonic kidney 293T cells. Transfection and purification of viral particles were performed as previously described (83, 84). Vector titers were determined using quantitative real-time PCR amplification as previously described (85). Endotoxin contamination of plasmids, crude vector lysates, and purified vectors was measured by Pyrotell-T kinetic turbidimetric assessment (Associates of Cape Cod) following the manufacturer’s protocols and found to be below 2.5 EU/ml.

### Intravitreal AAV injections

For testing the effectiveness of the vectors *in vivo*, 1 μl of a virus suspension containing over 5.0 × 10^11^ particles/ml of AAV2Cre or AAV2CreΔC was intravitreally injected into the eyes of adult mT/mG mice. For conditional knockout of mTOR, Raptor, and Rictor, the same strategy was used to inject *mTOR*^*f/f*^, *Rptor*^*f/f*^, and *Rctor*^*f/f*^ mice, respectively. While one eye received the Cre-expressing AAV, the contralateral eye received the CreΔC-expressing AAV. Briefly, to deliver the viruses, mice were anesthetized with an intraperitoneal injection of a ketamine (93 mg/kg) and xylazine (8 mg/kg) mixture and treated with topical proparacaine 0.5% (Alcon). Prior to injection, the conjunctiva was reflected, and a 32-gauge needle was used to puncture the sclera 1 mm below the limbus. A pulled borosilicate glass capillary connected to a Nanoject II microinjector (Drummond Scientific) was then used to slowly deliver 1 μl of virus suspension before applying an antibiotic eye ointment. While mT/mG mice eyes were recovered 6 weeks after injection, eyes from cKO were harvested at the durations specified for each analysis. mT/mG mice eyes were prepared and sectioned (10 μm thickness) as described previously and probed with the goat anti-tdTomato (1:500 dilution) and rabbit anti-GFP (1:200 dilution) antibodies and then secondary antibodies conjugated to AF488 or AF594 (Jackson Immunoresearch). Images were obtained using a Leica SP5 confocal microscope.

### Statistical analysis

All values are expressed as mean ± standard errors. Since we could not assume the normal distribution of the data, statistical analyses were conducted using unpaired nonparametric Mann–Whitney *U* tests using Prism (version 9.2.0; GraphPad Software, Inc). Differences were considered significant with a *p* value ≤0.05.

## Data availability

All data are contained within the article.

## Supporting information

This article contains supporting information.

## Conflict of interest

The authors declare that they have no conflicts of interest with the contents of this article.

## Supporting information

JBC Final Data Supplement(new)-041322

## References

[1] Fu Z., Sun Y., Cakir B., Tomita Y., Huang S., Wang Z., Liu C.H., S S.C., Britton W., T S.K., Antonetti D.A., Hellstrom A., E H Smith L. (2020). Targeting neurovascular interaction in retinal disorders. Int. J. Mol. Sci..

[2] Metea M.R., Newman E.A. (2007). Signalling within the neurovascular unit in the mammalian retina. Exp. Physiol..

[3] Usui Y., Westenskow P.D., Kurihara T., Aguilar E., Sakimoto S., Paris L.P., Wittgrove C., Feitelberg D., Friedlander M.S., Moreno S.K., Dorrell M.I., Friedlander M. (2015). Neurovascular crosstalk between interneurons and capillaries is required for vision. J. Clin. Invest..

[4] Newman E.A. (2013). Functional hyperemia and mechanisms of neurovascular coupling in the retinal vasculature. J. Cereb. Blood Flow Metab..

[5] Metea M.R., Newman E.A. (2006). Glial cells dilate and constrict blood vessels: A mechanism of neurovascular coupling. J. Neurosci..

[6] Wangsa-Wirawan N.D., Linsenmeier R.A. (2003). Retinal oxygen: Fundamental and clinical aspects. Arch. Ophthalmol..

[7] Fort P.E., Losiewicz M.K., Pennathur S., Jefferson L.S., Kimball S.R., Abcouwer S.F., Gardner T.W. (2014). mTORC1-independent reduction of retinal protein synthesis in type 1 diabetes. Diabetes.

[8] Gardner T.W., Abcouwer S.F., Losiewicz M.K., Fort P.E. (2015). Phosphatase control of 4E-BP1 phosphorylation state is central for glycolytic regulation of retinal protein synthesis. Am. J. Physiol. Endocrinol. Metab..

[9] Chihara E. (1981). Impairment of protein synthesis in the retinal tissue in diabetic rabbits: Secondary reduction of fast axonal transport. J. Neurochem..

[10] Chihara E., Sakugawa M., Entani S. (1982). Reduced protein synthesis in diabetic retina and secondary reduction of slow axonal transport. Brain Res..

[11] Wong-Riley M.T. (2010). Energy metabolism of the visual system. Eye Brain.

[12] Rajala R.V.S. (2020). Aerobic glycolysis in the retina: Functional roles of pyruvate kinase isoforms. Front. Cell Dev. Biol..

[13] Lindsay K.J., Du J., Sloat S.R., Contreras L., Linton J.D., Turner S.J., Sadilek M., Satrústegui J., Hurley J.B. (2014). Pyruvate kinase and aspartate-glutamate carrier distributions reveal key metabolic links between neurons and glia in retina. Proc. Natl. Acad. Sci. U. S. A..

[14] Léveillard T., Sahel J.A. (2017). Metabolic and redox signaling in the retina. Cell Mol. Life Sci..

[15] Karlsson J.O., Sjostrand J. (1971). Synthesis, migration and turnover of protein in retinal ganglion cells. J. Neurochem..

[16] Leon J.A., Britt J.M., Hopp R.H., Mills R.P., Milam A.H. (1990). Effects of fluorouracil and fluorouridine on protein synthesis in rabbit retina. Invest. Ophthalmol. Vis. Sci..

[17] Steinman L., Ames A. (1974). The sites of synthesis and the subsequent migration of newly synthesized protein in retina. Tissue Cell.

[18] Saxton R.A., Sabatini D.M. (2017). mTOR signaling in growth, metabolism, and disease. Cell.

[19] Boutouja F., Stiehm C.M., Platta H.W. (2019). mTOR: A cellular regulator interface in health and disease. Cells.

[20] Kim J., Guan K.L. (2019). mTOR as a central hub of nutrient signalling and cell growth. Nat. Cell Biol..

[21] Jhanwar-Uniyal M., Amin A.G., Cooper J.B., Das K., Schmidt M.H., Murali R. (2017). Discrete signaling mechanisms of mTORC1 and mTORC2: Connected yet apart in cellular and molecular aspects. Adv. Biol. Regul..

[22] Xie J., Proud C.G. (2014). Signaling crosstalk between the mTOR complexes. Translation (Austin).

[23] Switon K., Kotulska K., Janusz-Kaminska A., Zmorzynska J., Jaworski J. (2017). Molecular neurobiology of mTOR. Neuroscience.

[24] Tee A.R., Sampson J.R., Pal D.K., Bateman J.M. (2016). The role of mTOR signalling in neurogenesis, insights from tuberous sclerosis complex. Semin. Cell Dev. Biol..

[25] Garza-Lombo C., Gonsebatt M.E. (2016). Mammalian target of rapamycin: Its role in early neural development and in adult and aged brain function. Front. Cell Neurosci..

[26] Park K.K., Liu K., Hu Y., Smith P.D., Wang C., Cai B., Xu B., Connolly L., Kramvis I., Sahin M., He Z. (2008). Promoting axon regeneration in the adult CNS by modulation of the PTEN/mTOR pathway. Science.

[27] Jones I., Hagglund A.C., Tornqvist G., Nord C., Ahlgren U., Carlsson L. (2015). A novel mouse model of tuberous sclerosis complex (TSC): Eye-specific Tsc1-ablation disrupts visual-pathway development. Dis. Model. Mech..

[28] Choi J.H., Jo H.S., Lim S., Kim H.T., Lee K.W., Moon K.H., Ha T., Kwak S.S., Kim Y., Lee E.J., Joe C.O., Kim J.W. (2018). mTORC1 accelerates retinal development *via* the immunoproteasome. Nat. Commun..

[29] Ma S., Venkatesh A., Langellotto F., Le Y.Z., Hall M.N., Ruegg M.A., Punzo C. (2015). Loss of mTOR signaling affects cone function, cone structure and expression of cone specific proteins without affecting cone survival. Exp. Eye Res..

[30] Petit L., Punzo C. (2015). mTORC1 sustains vision in retinitis pigmentosa. Oncotarget.

[31] Zhang J., Yang D., Huang H., Sun Y., Hu Y. (2018). Coordination of necessary and permissive signals by PTEN inhibition for CNS axon regeneration. Front. Neurosci..

[32] Losiewicz M.K., Elghazi L., Fingar D.C., Rajala R.V.S., Lentz S.I., Fort P.E., Abcouwer S.F., Gardner T.W. (2020). mTORC1 and mTORC2 expression in inner retinal neurons and glial cells. Exp. Eye Res..

[33] Schmidt E.K., Clavarino G., Ceppi M., Pierre P. (2009). SUnSET, a nonradioactive method to monitor protein synthesis. Nat. Methods.

[34] Goodman C.A., Hornberger T.A. (2013). Measuring protein synthesis with SUnSET: A valid alternative to traditional techniques?. Exerc. Sport Sci. Rev..

[35] Kelleher A.R., Kimball S.R., Dennis M.D., Schilder R.J., Jefferson L.S. (2013). The mTORC1 signaling repressors REDD1/2 are rapidly induced and activation of p70S6K1 by leucine is defective in skeletal muscle of an immobilized rat hindlimb. Am. J. Physiol. Endocrinol. Metab..

[36] Starr C.R., Nyankerh C.N.A., Qi X., Hu Y., Gorbatyuk O.S., Sonenberg N., Boulton M.E., Gorbatyuk M.S. (2019). Role of translational attenuation in inherited retinal degeneration. Invest. Ophthalmol. Vis. Sci..

[37] Starr C.R., Pitale P.M., Gorbatyuk M. (2018). Translational attenuation and retinal degeneration in mice with an active integrated stress response. Cell Death Dis..

[38] Su B., Jacinto E. (2011). Mammalian TOR signaling to the AGC kinases. Crit. Rev. Biochem. Mol. Biol..

[39] Ha Y., Liu W., Liu H., Zhu S., Xia F., Gerson J.E., Azhar N.A., Tilton R.G., Motamedi M., Kayed R., Zhang W. (2018). AAV2-mediated GRP78 transfer alleviates retinal neuronal injury by downregulating ER stress and Tau oligomer formation. Invest. Ophthalmol. Vis. Sci..

[40] Maraini G., Franguelli R. (1962). Protein metabolism of rabbit retina *in vitro* studied by means of high resolution autoradiography. Ophthalmologica.

[41] Maraini G., Franguelli R. (1962). Radioautographic investigations on nucleic acids and protein metabolism of the retina *in vitro*. Ophthalmologica.

[42] Nover A., Schultze B. (1960). [Autoradiographic study of protein metabolism in the tissues and cells of the eye. (Studied with S-35-thioamino acids. C-14-amino acids, H-3-leucine in the mouse, rat and rabbit)]. Albrecht Von Graefes Arch. Ophthalmol..

[43] Schultze B., Oehlert W., Maurer W. (1959). [Autoradiographic study on the mechanism of new protein formation in ganglion cells; studies with S35 thioamino acid in rabbits]. Beitr Pathol. Anat..

[44] Hodson S., Marshall J. (1967). Tyrosine incorporation into the rabbit retina. J. Cell Biol..

[45] Kimball S.R., Farrell P.A., Jefferson L.S. (2002). Invited Review: Role of insulin in translational control of protein synthesis in skeletal muscle by amino acids or exercise. J. Appl. Physiol..

[46] Dennis M.D., Baum J.I., Kimball S.R., Jefferson L.S. (2011). Mechanisms involved in the coordinate regulation of mTORC1 by insulin and amino acids. J. Biol. Chem..

[47] Maus M., Torrens Y., Gauchy C., Bretin S., Nairn A.C., Glowinski J., Premont J. (2006). 2-Deoxyglucose and NMDA inhibit protein synthesis in neurons and regulate phosphorylation of elongation factor-2 by distinct mechanisms. J. Neurochem..

[48] Kurtoglu M., Maher J.C., Lampidis T.J. (2007). Differential toxic mechanisms of 2-deoxy-D-glucose *versus* 2-fluorodeoxy-D-glucose in hypoxic and normoxic tumor cells. Antioxid. Redox Signal..

[49] Tailler M., Lindqvist L.M., Gibson L., Adams J.M. (2018). By reducing global mRNA translation in several ways, 2-deoxyglucose lowers MCL-1 protein and sensitizes hemopoietic tumor cells to BH3 mimetic ABT737. Cell Death Differ..

[50] Umegaki Y., Brotons A.M., Nakanishi Y., Luo Z., Zhang H., Bonni A., Ikeuchi Y. (2018). Palladin is a neuron-specific translational target of mTOR signaling that regulates axon morphogenesis. J. Neurosci..

[51] Thoreen C.C., Chantranupong L., Keys H.R., Wang T., Gray N.S., Sabatini D.M. (2012). A unifying model for mTORC1-mediated regulation of mRNA translation. Nature.

[52] Thoreen C.C. (2017). The molecular basis of mTORC1-regulated translation. Biochem. Soc. Trans..

[53] Jones I., Hagglund A.C., Carlsson L. (2019). Reduced mTORC1-signalling in retinal progenitor cells leads to visual pathway dysfunction. Biol. Open.

[54] Shigeoka T., Lu B., Holt C.E. (2013). Cell biology in neuroscience: RNA-based mechanisms underlying axon guidance. J. Cell Biol..

[55] Koley S., Rozenbaum M., Fainzilber M., Terenzio M. (2019). Translating regeneration: Local protein synthesis in the neuronal injury response. Neurosci. Res..

[56] Shigeoka T., Jung H., Jung J., Turner-Bridger B., Ohk J., Lin J.Q., Amieux P.S., Holt C.E. (2016). Dynamic axonal translation in developing and mature visual circuits. Cell.

[57] Shah S.H., Goldberg J.L. (2018). The role of axon transport in neuroprotection and regeneration. Dev. Neurobiol..

[58] Schiapparelli L.M., Shah S.H., Ma Y., McClatchy D.B., Sharma P., Li J., Yates J.R., Goldberg J.L., Cline H.T. (2019). The retinal ganglion cell transportome identifies proteins transported to axons and presynaptic compartments in the visual system *in vivo*. Cell Rep..

[59] Zieger M., Punzo C. (2016). Improved cell metabolism prolongs photoreceptor survival upon retinal-pigmented epithelium loss in the sodium iodate induced model of geographic atrophy. Oncotarget.

[60] Kwong J.M., Caprioli J., Piri N. (2010). RNA binding protein with multiple splicing: A new marker for retinal ganglion cells. Invest. Ophthalmol. Vis. Sci..

[61] Masin L., Claes M., Bergmans S., Cools L., Andries L., Davis B.M., Moons L., De Groef L. (2021). A novel retinal ganglion cell quantification tool based on deep learning. Sci. Rep..

[62] Rodriguez A.R., de Sevilla Müller L.P., Brecha N.C. (2014). The RNA binding protein RBPMS is a selective marker of ganglion cells in the mammalian retina. J. Comp. Neurol..

[63] Saha S., Greferath U., Vessey K.A., Grayden D.B., Burkitt A.N., Fletcher E.L. (2016). Changes in ganglion cells during retinal degeneration. Neuroscience.

[64] Lam C., Li K.K., Do C.W., Chan H., To C.H., Kwong J.M.K. (2019). Quantitative profiling of regional protein expression in rat retina after partial optic nerve transection using fluorescence difference two-dimensional gel electrophoresis. Mol. Med. Rep..

[65] Romano G.L., Amato R., Lazzara F., Porciatti V., Chou T.H., Drago F., Bucolo C. (2020). P2X7 receptor antagonism preserves retinal ganglion cells in glaucomatous mice. Biochem. Pharmacol..

[66] Farazi T.A., Leonhardt C.S., Mukherjee N., Mihailovic A., Li S., Max K.E., Meyer C., Yamaji M., Cekan P., Jacobs N.C., Gerstberger S., Bognanni C., Larsson E., Ohler U., Tuschl T. (2014). Identification of the RNA recognition element of the RBPMS family of RNA-binding proteins and their transcriptome-wide mRNA targets. RNA.

[67] Hörnberg H., Cioni J.M., Harris W.A., Holt C.E. (2016). Hermes regulates axon sorting in the optic tract by post-trancriptional regulation of neuropilin 1. J. Neurosci..

[68] Hörnberg H., Wollerton-van Horck F., Maurus D., Zwart M., Svoboda H., Harris W.A., Holt C.E. (2013). RNA-binding protein Hermes/RBPMS inversely affects synapse density and axon arbor formation in retinal ganglion cells *in vivo*. J. Neurosci..

[69] Pereiro X., Ruzafa N., Urcola J.H., Sharma S.C., Vecino E. (2020). Differential distribution of RBPMS in pig, rat, and human retina after damage. Int. J. Mol. Sci..

[70] Muzumdar M.D., Tasic B., Miyamichi K., Li L., Luo L. (2007). A global double-fluorescent Cre reporter mouse. Genesis.

[71] Risson V., Mazelin L., Roceri M., Sanchez H., Moncollin V., Corneloup C., Richard-Bulteau H., Vignaud A., Baas D., Defour A., Freyssenet D., Tanti J.F., Le-Marchand-Brustel Y., Ferrier B., Conjard-Duplany A. (2009). Muscle inactivation of mTOR causes metabolic and dystrophin defects leading to severe myopathy. J. Cell Biol..

[72] Polak P., Cybulski N., Feige J.N., Auwerx J., Ruegg M.A., Hall M.N. (2008). Adipose-specific knockout of raptor results in lean mice with enhanced mitochondrial respiration. Cell Metab..

[73] Seigel G.M. (2014). Review: R28 retinal precursor cells: The first 20 years. Mol. Vis..

[74] Sindelar D.K., Ste Marie L., Miura G.I., Palmiter R.D., McMinn J.E., Morton G.J., Schwartz M.W. (2004). Neuropeptide Y is required for hyperphagic feeding in response to neuroglucopenia. Endocrinology.

[75] Schindelin J., Arganda-Carreras I., Frise E., Kaynig V., Longair M., Pietzsch T., Preibisch S., Rueden C., Saalfeld S., Schmid B., Tinevez J.Y., White D.J., Hartenstein V., Eliceiri K., Tomancak P. (2012). Fiji: An open-source platform for biological-image analysis. Nat. Methods.

[76] Elghazi L., Blandino-Rosano M., Alejandro E., Cras-Meneur C., Bernal-Mizrachi E. (2017). Role of nutrients and mTOR signaling in the regulation of pancreatic progenitors development. Mol. Metab..

[77] Zuiderveld K., Heckbert P.S. (1994). Graphics Gems IV.

[78] Shimshek D.R., Kim J., Hübner M.R., Spergel D.J., Buchholz F., Casanova E., Stewart A.F., Seeburg P.H., Sprengel R. (2002). Codon-improved Cre recombinase (iCre) expression in the mouse. Genesis.

[79] Hartung M., Kisters-Woike B. (1998). Cre mutants with altered DNA binding properties. J. Biol. Chem..

[80] Guggenhuber S., Monory K., Lutz B., Klugmann M. (2010). AAV vector-mediated overexpression of CB1 cannabinoid receptor in pyramidal neurons of the hippocampus protects against seizure-induced excitoxicity. PLoS One.

[81] Assouvie A., Daley-Bauer L.P., Rousselet G. (2018). Growing murine bone marrow-derived macrophages. Methods Mol. Biol..

[82] Xu J. (2005). Preparation, culture, and immortalization of mouse embryonic fibroblasts. Curr. Protoc. Mol. Biol..

[83] Nishiguchi K.M., Carvalho L.S., Rizzi M., Powell K., Holthaus S.M., Azam S.A., Duran Y., Ribeiro J., Luhmann U.F., Bainbridge J.W., Smith A.J., Ali R.R. (2015). Gene therapy restores vision in rd1 mice after removal of a confounding mutation in Gpr179. Nat. Commun..

[84] Georgiadis A., Duran Y., Ribeiro J., Abelleira-Hervas L., Robbie S.J., Sünkel-Laing B., Fourali S., Gonzalez-Cordero A., Cristante E., Michaelides M., Bainbridge J.W., Smith A.J., Ali R.R. (2016). Development of an optimized AAV2/5 gene therapy vector for Leber congenital amaurosis owing to defects in RPE65. Gene Ther..

[85] Aurnhammer C., Haase M., Muether N., Hausl M., Rauschhuber C., Huber I., Nitschko H., Busch U., Sing A., Ehrhardt A., Baiker A. (2012). Universal real-time PCR for the detection and quantification of adeno-associated virus serotype 2-derived inverted terminal repeat sequences. Hum. Gene Ther. Methods.

