# Supplementary material for: mTORC1 regulates high levels of protein synthesis in retinal ganglion cells of adult mice
Source: J Biol Chem. 2022 Apr 18;298(6):101944. doi: 10.1016/j.jbc.2022.101944 (PMC9117545; doi:10.1016/j.jbc.2022.101944)
Supplement: JBC Final Data Supplement(new)-041322 [file mmc1.pdf]

SUPPLEMENTAL DATA

mTORC1 Regulates High Levels of Protein Synthesis in Retinal Ganglion Cells of Adult Mice

[1,2]Patrice E. Fort
[1]Mandy K. Losiewicz
[1]Lynda Elghazi
[1]Dejuan Kong
[3]Corentin Cras-Méneur
[4]Diane C. Fingar
[5]Scot R. Kimball
[6]Raju V.S. Rajala
[7]Alexander J. Smith
[1,7]Robin R Ali
[1]Steven F. Abcouwer*
[1,2,3]Thomas W. Gardner

Supplemental Figure legends

Supplemental Data Figure S1: **Effect of puromycin dosage on its incorporation into mouse retinas. (A)** A representative western blot of whole mouse retina lysates harvested 30 minutes after intraperitoneal injection with puromycin at 0, 100, 400, and 800 mg/kg. **(B)** Quantification of western blot data in (A). Data are shown as mean ± SD.

Supplemental Data Figure S2: **Dot-blot SUnSET assay. (A)** Effect of protein loading on dot blot SUnSET assay using retinal lysates from mice treated with 100 and 400 mg/kg puromycin. **(B)** Quantification of the dot blot intensities showing best-fit linear correlations of data from 1 to 10 μg/well.

Supplemental Data Figure S3: Treatment of mice with 2-deoxyglucose does not diminish retinal mTORC1 activity as indicated by ribosomal S6 phosphorylation. (A) Representative images of pS6 IF (S240/S244, green) RBPMS IF (red) and Hoechst staining of nuclei (blue) in retinal sections of mice with and without 2-DG treatment at 30 min prior to systemic puromycin administration (as shown in Figure 4). (B) Quantification of pS6 IF intensity in the GCL. Data are shown as mean ± SEM, n=8/group.

Supplemental Data Figure S4: ***In vitro* testing of the novel control vector expressing a mutant Cre (CreΔC) with no recombinase activity**. *In vitro* testing of CreΔC recombination by transfection of pD10.CMV.iCre or pD10.CMV.iCreΔC into primary MEF **(A)** and BMDMϕ **(B)** cells obtained from mT/mG reporter mice containing a transgene with loxP-flanked membrane-targeted tdTomato sequence followed a coding sequence for membrane-tagged GFP sequence. GFP expression at 3 day (MEF) or 7 day (BMDMϕ) shows recombination in cells transfected with the Cre plasmid. Note that no recombination was detected in cells transfected with the CreΔC plasmid.

Supplemental Data Figure S5: Similar effect of conditional knockout of mTOR on protein synthesis in the GCL at 25 wk. (A) Representative *in situ* SUnSET assay and mTOR IF probing of retinal sections of *mtor*$^{f/f}$ mice 25 wk after intravitreal injection of AAV-Cre∆C (top rows) or AAV-Cre (bottom rows). Puromycinylated protein IF is shown in green, mTOR IF is shown in red, and Hoechst staining of nuclei is shown in blue. **(B)** Quantification of mTOR IF intensity in the GCL of retinas at 25 wk. Intensities were normalized to the mean values of those in the control (AAV-Cre∆C) retinas. **(C)** Quantification of puromycin incorporation in the GCL at 25 wk. Data are shown as mean ± SEM, n=6/group. **p ≤ 0.01 by Mann-Whitney u-test.

Supplemental Data Figure S6: Similar effect of conditional knockout of RAPTOR on protein synthesis in the GCL at 25 wk. (A) Representative *in situ* SUnSET assay and RAPTOR IF probing of retinal sections of *rptor*$^{f/f}$ mice at 25 wk after intravitreal injection of AAV-Cre∆C (top rows) or AAV-Cre (bottom rows). Puromycinylated protein IF is shown in green, Raptor IF is shown in red, and Hoechst staining of nuclei is shown in blue. **(B)** Quantification of Raptor IF intensity in the GCL of retinas at 25 wk. Intensities were normalized to the mean values of those in the control (AAV-Cre∆C) retinas. **(C)** Quantification of puromycin incorporation in the GCL at 25 wk. Data are shown as mean ± SEM, n=6/group. **p ≤ 0.01 by Mann-Whitney u-test.

Supplemental Data Figure S7: **Conditional knockout of RICTOR does not affect protein synthesis in the GCL. (A and D)** Representative *in situ* SUnSET assay and RICTOR IF probing of retinal sections of *rctor*$^{f/f}$ mice at 17 wk **(A)** or 25 wk **(D)** after intravitreal injection of AAV-Cre∆C (top rows) or AAV-Cre (bottom rows). Puromycinylated protein IF is shown in green, RICTOR IF is shown in red, and Hoechst staining of nuclei is shown in blue. **(B and E)** Quantification of RICTOR IF intensity in the GCL of retinas at 17 wk **(B)** or 25 wk **(E)**. Intensities were normalized to the mean values of those in the control (AAV-

CreΔC) retinas. **(C and F)** Quantification of puromycin incorporation in the GCL at 17 wk **(C)** or 25 wk **(F)**. Data are shown as mean ± SEM, n=6/group. **p ≤ 0.01 by Mann-Whitney u-test.

Supplemental Data Figure S8: Effect of conditional knockout of mTOR on phosphorylation of S6 ribosomal protein and expression of RBPMS in the GCL at 25 wk. (A) Representative pS6 (S240/S244) and RBPMS IF in retinal sections of *mtor*$^{f/f}$ mice at 25 wk after intravitreal injection of AAV-CreΔC (top rows) or AAV-Cre (bottom rows). pS6 IF is shown in green, RBPMS IF is shown in red, and Hoechst staining of nuclei is shown in blue. **(B)** Quantification of pS6 IF intensity in the GCL of retinas at 25 wk. **(C)** Quantification of RBPMS IF intensity in the GCL of retinas at 25 wk. **(D)** Percentage of RBPMS-positive soma in GCL of retinas at 25 wk. **(E)** Total number of cell nuclei per image in the GCL of retinas at 25 wk. Data are shown as mean ± SEM, n=7/group. ** p ≤ 0.01, *** p ≤ 0.001.by Mann-Whitney test.

Supplemental Data Figure S9: Similar effect of conditional knockout of RAPTOR on phosphorylation of S6 ribosomal protein and expression of RBPMS in the GCL at 25 wk. (A) Representative pS6 (S240/S244) and RBPMS IF in retinal sections of *rptor*$^{f/f}$ mice at 25 wk after intravitreal injection of AAV-CreΔC (top rows) or AAV-Cre (bottom rows). pS6 IF is shown in green, RBPMS IF is shown in red, and Hoechst staining of nuclei is shown in blue. **(B)** Quantification of pS6 IF intensity in the GCL of retinas at 25 wk. **(C)** Quantification of RBPMS IF intensity in the GCL of retinas at 25 wk. **(D)** Percentage of RBPMS-positive soma in GCL of retinas at 25 wk. **(E)** Total number of cell nuclei per image in the GCL of retinas at 25 wk. Data are shown as mean ± SEM, n=6/group. ** p ≤ 0.01, *** p ≤ 0.001 by Mann-Whitney test.

A

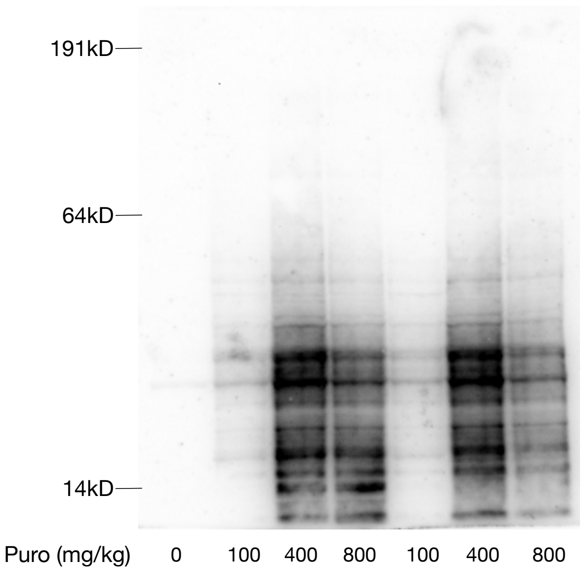

B

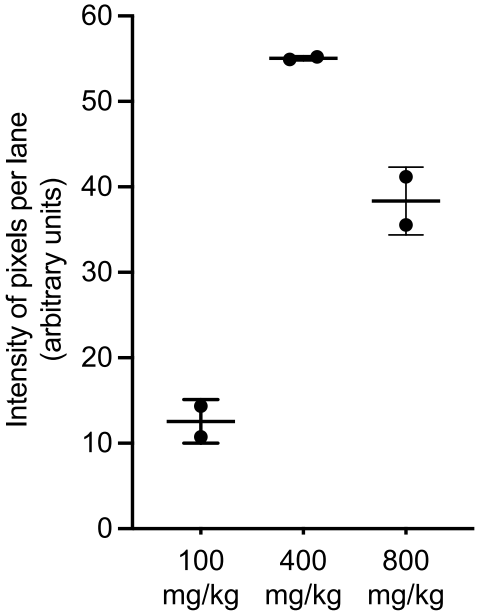

Supplemental Figure S1

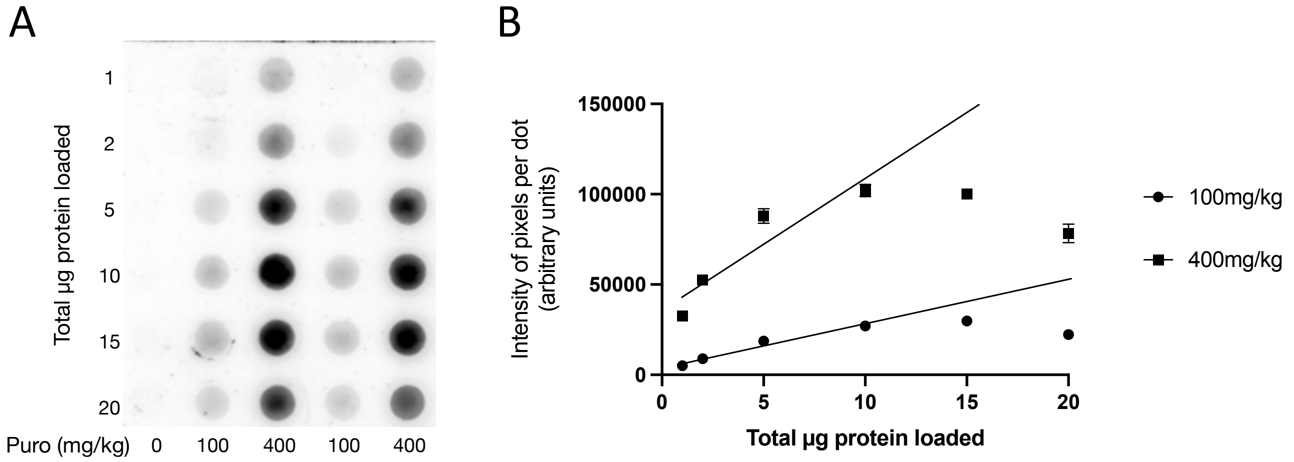

Supplemental Figure S2

A

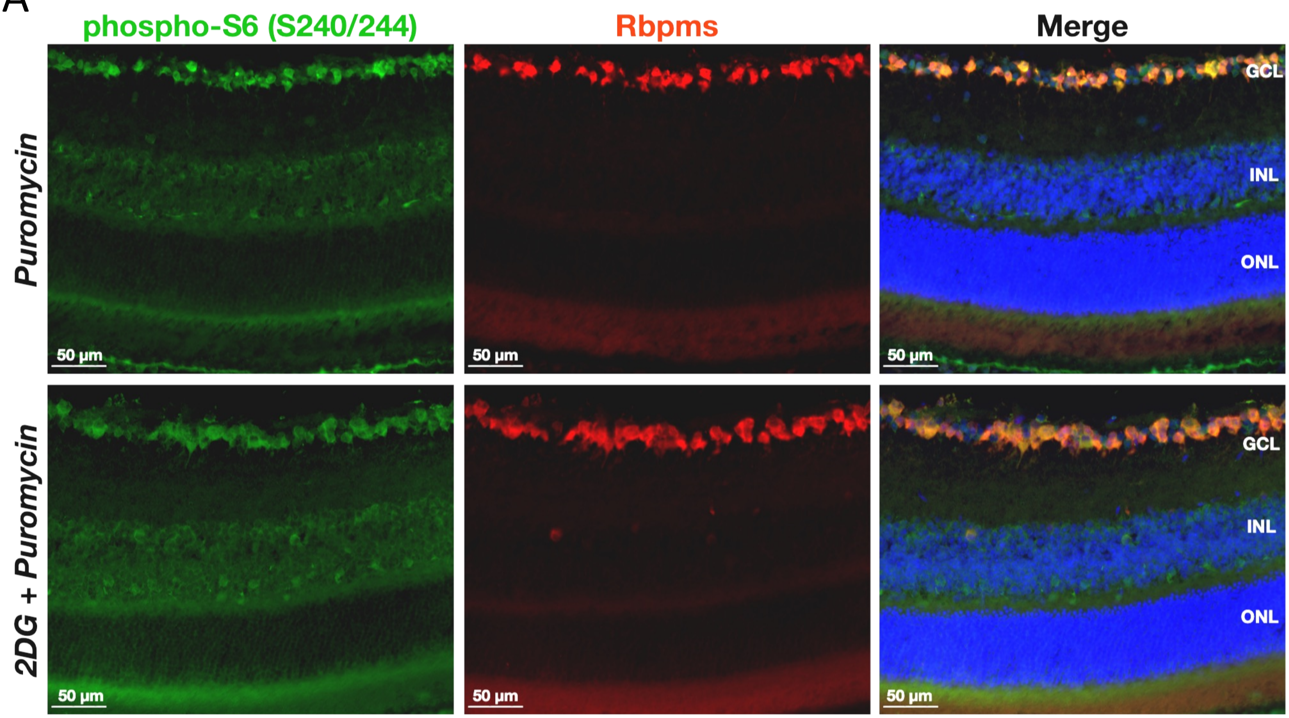

B

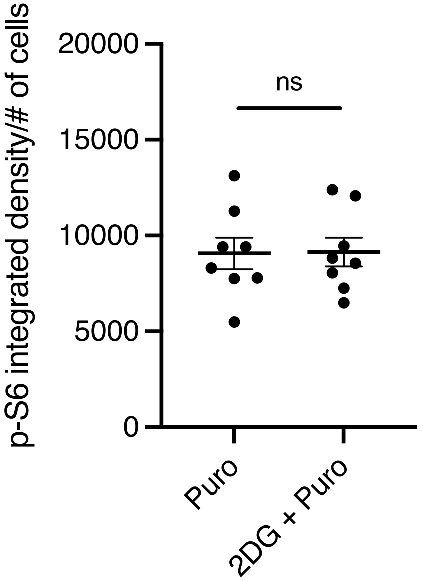

Supplemental Figure S3

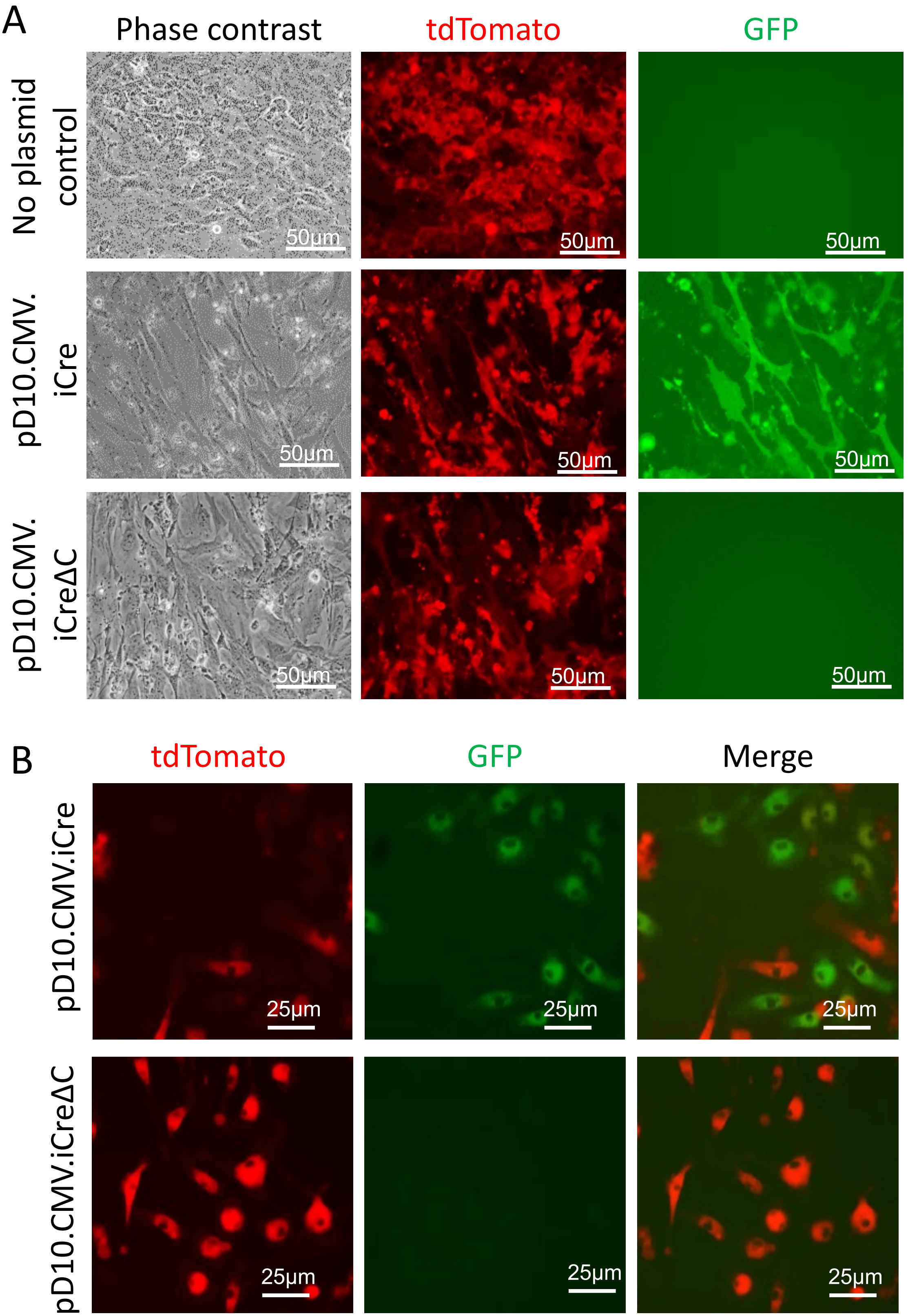

Supplemental Figure S4

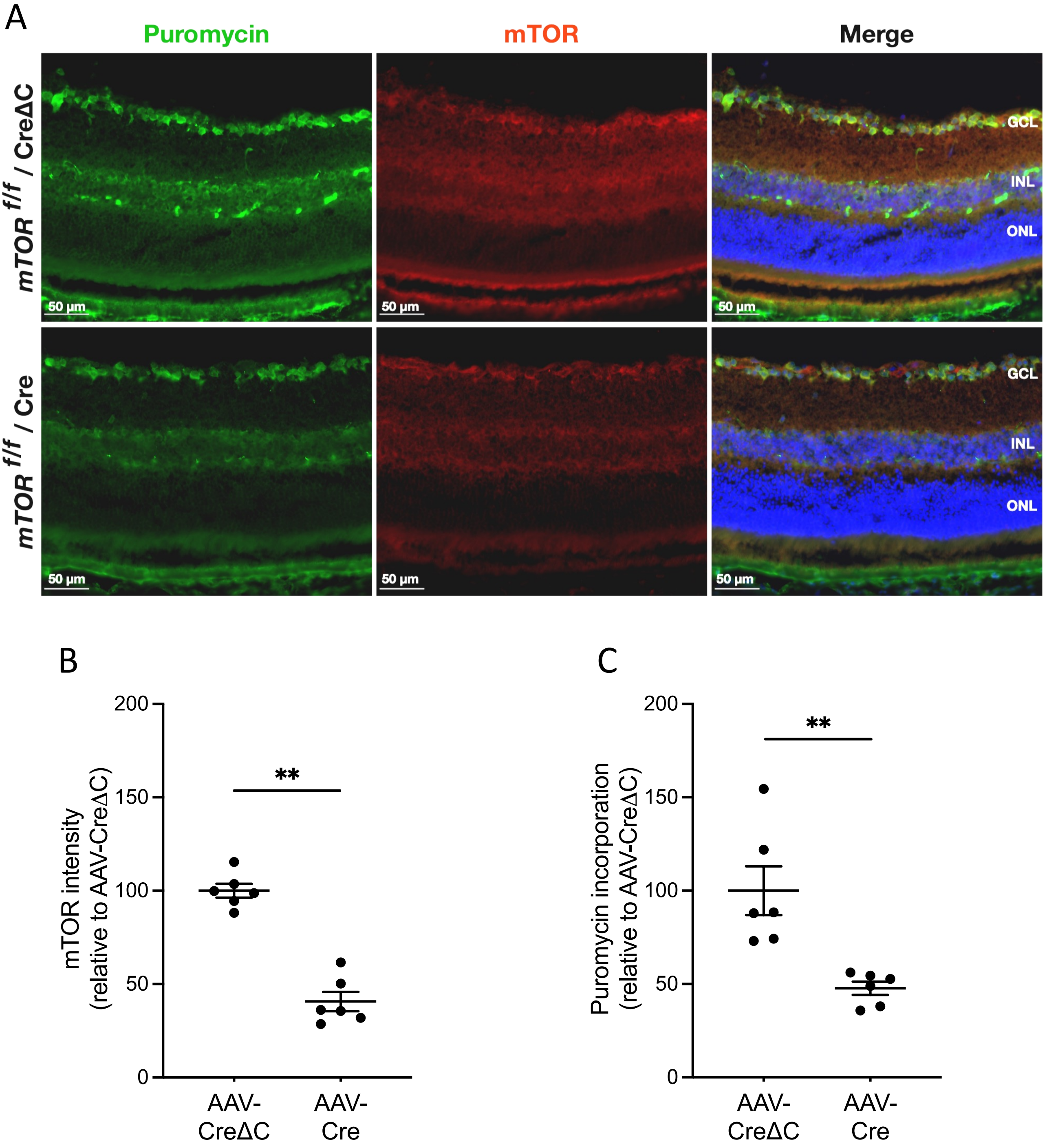

Supplemental Figure S5

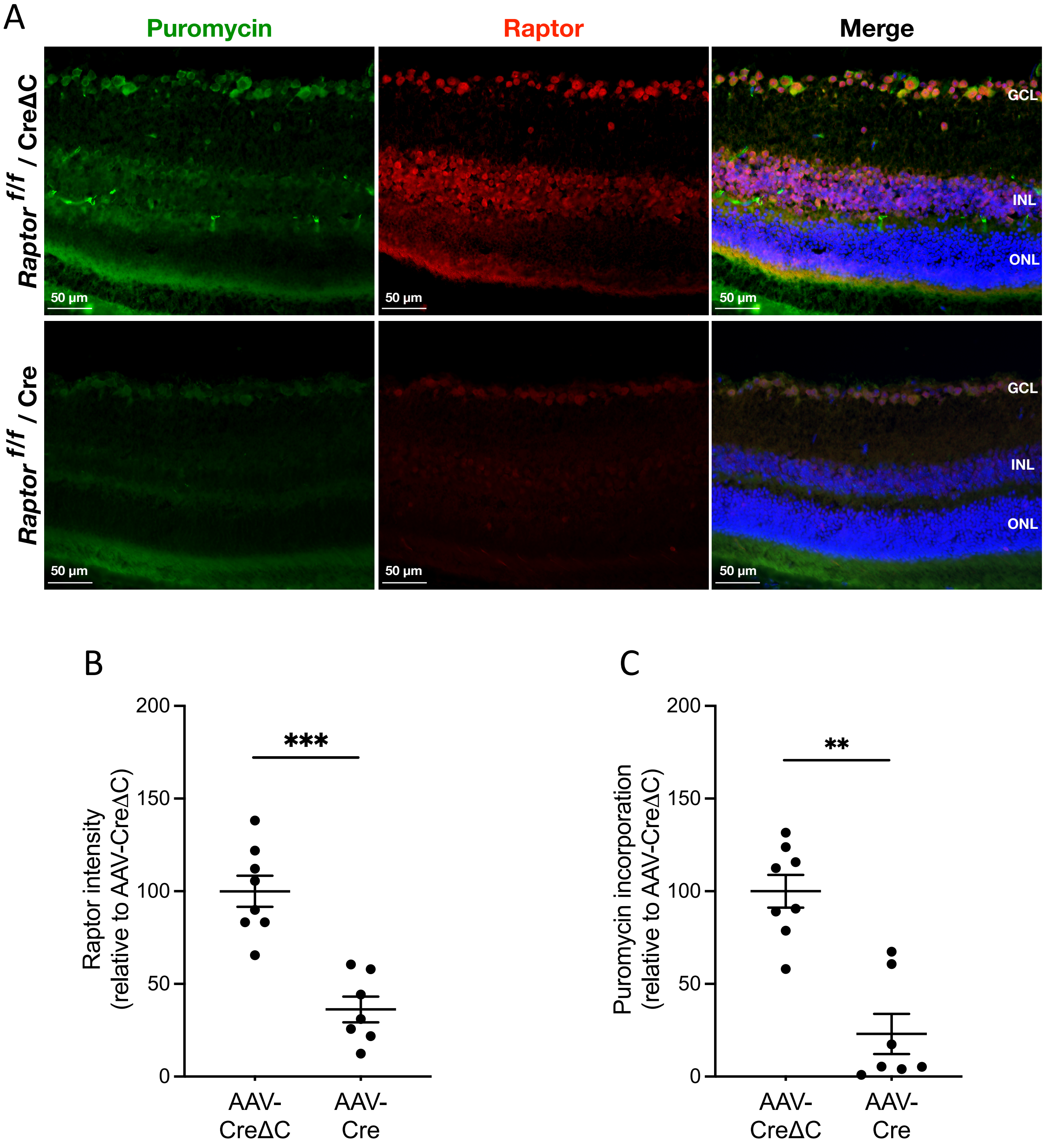

Supplemental Figure S6

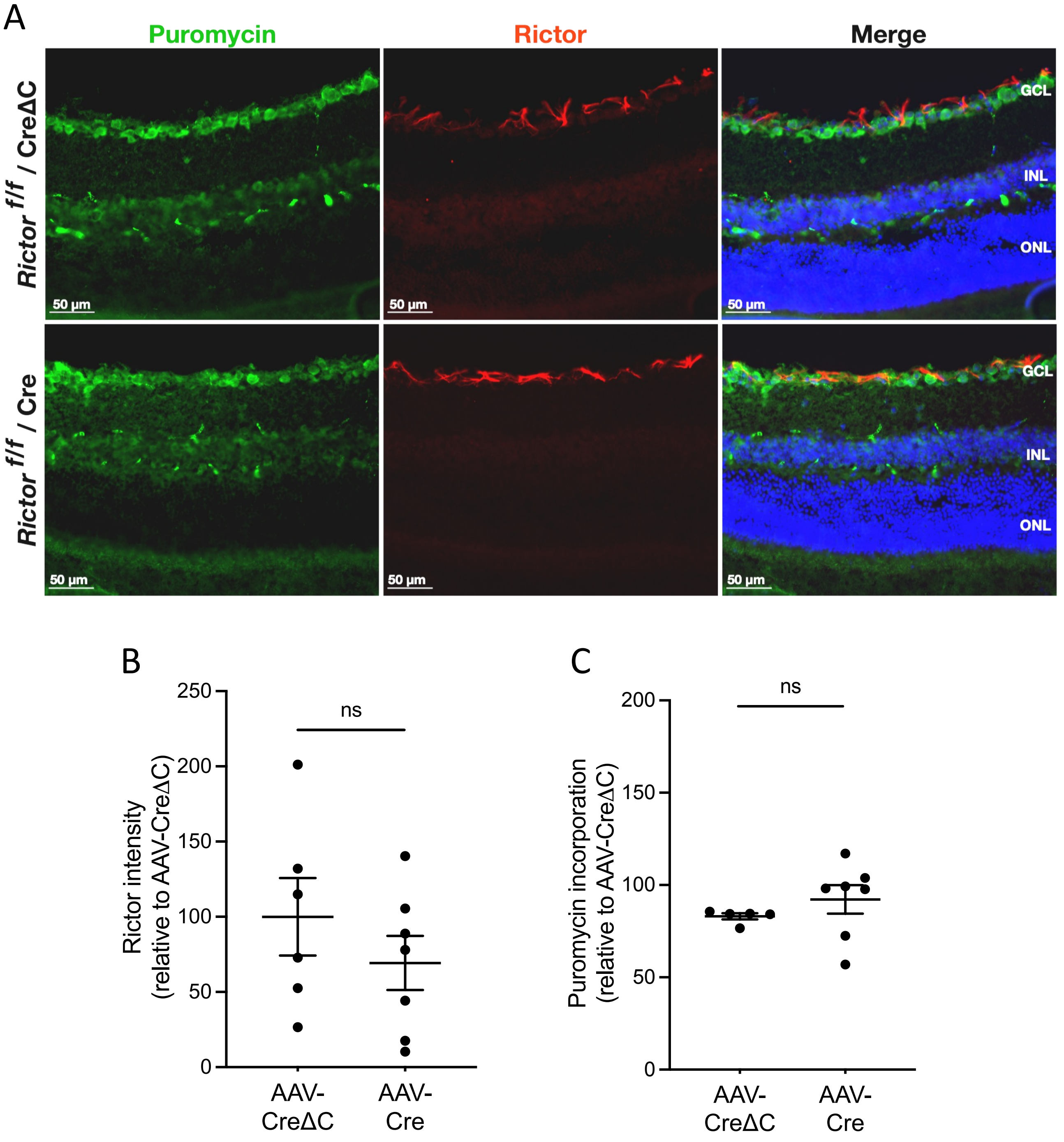

Supplemental Figure S7

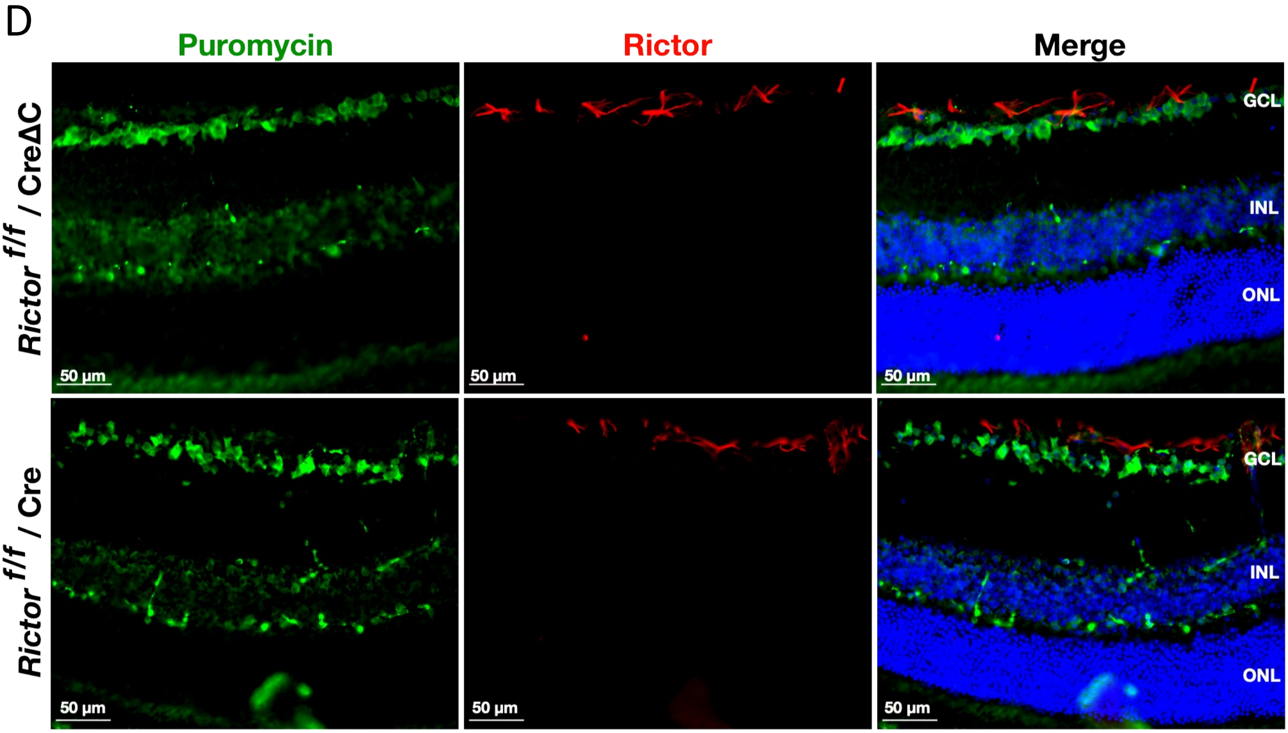

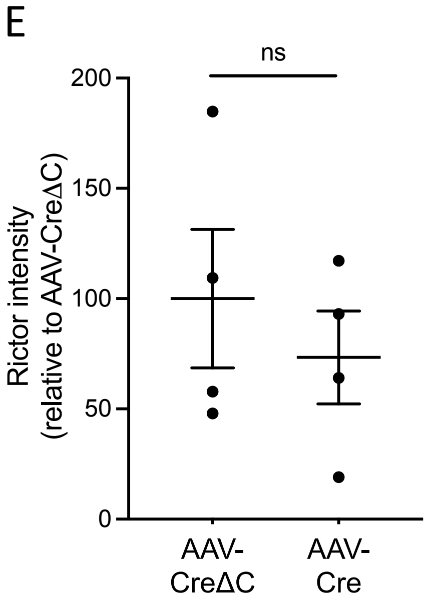

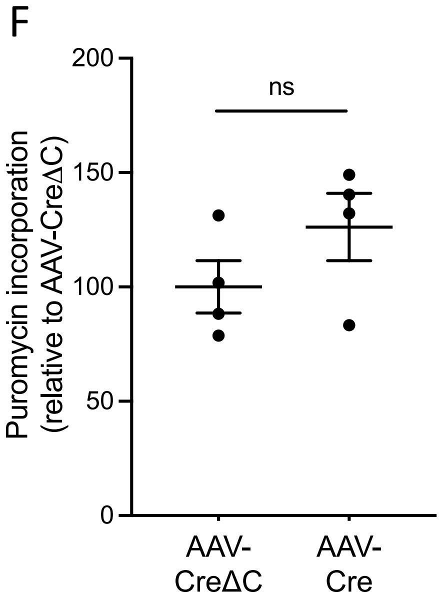

Supplemental Figure S7 (continued)

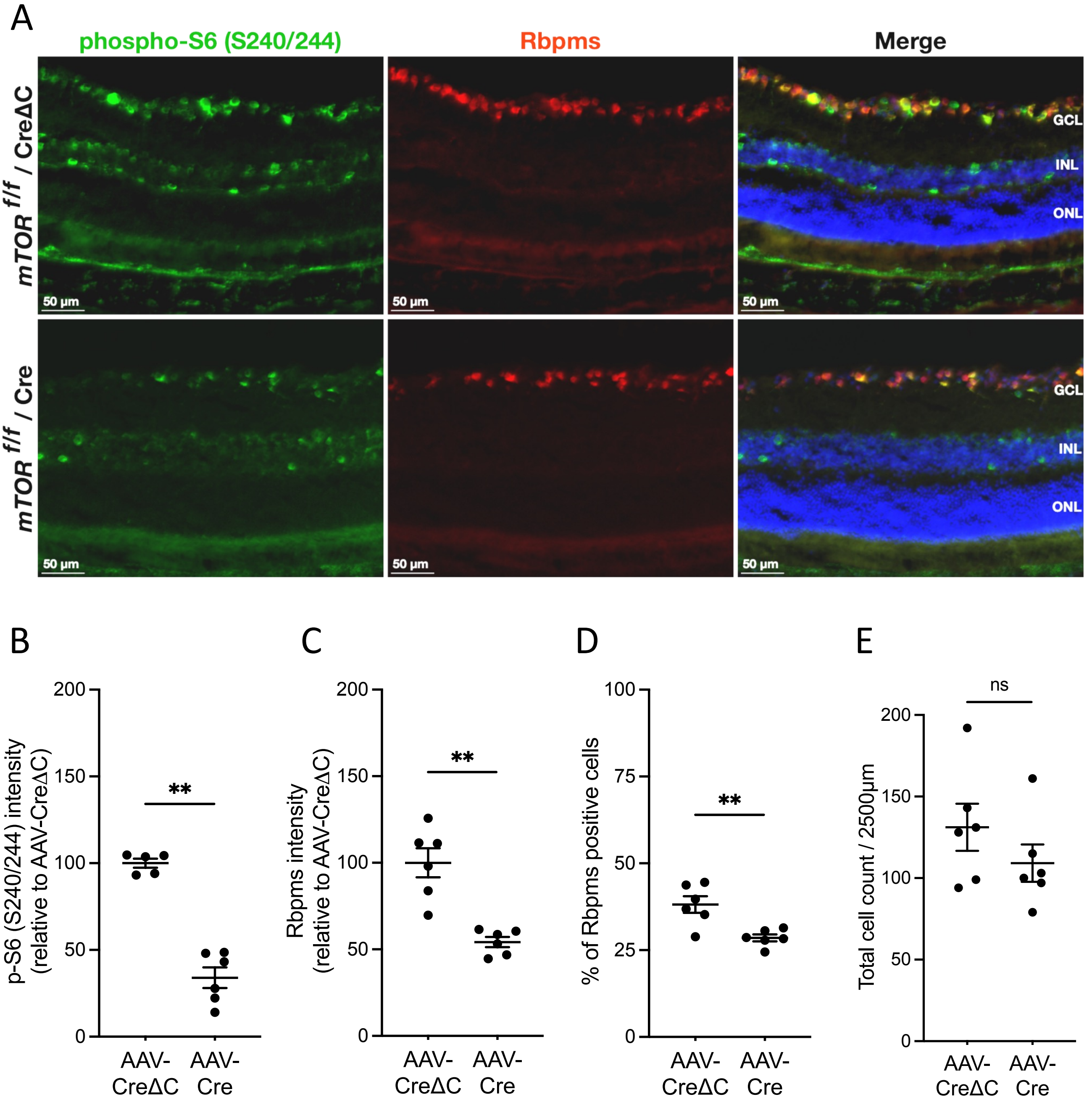

Supplemental Figure S8

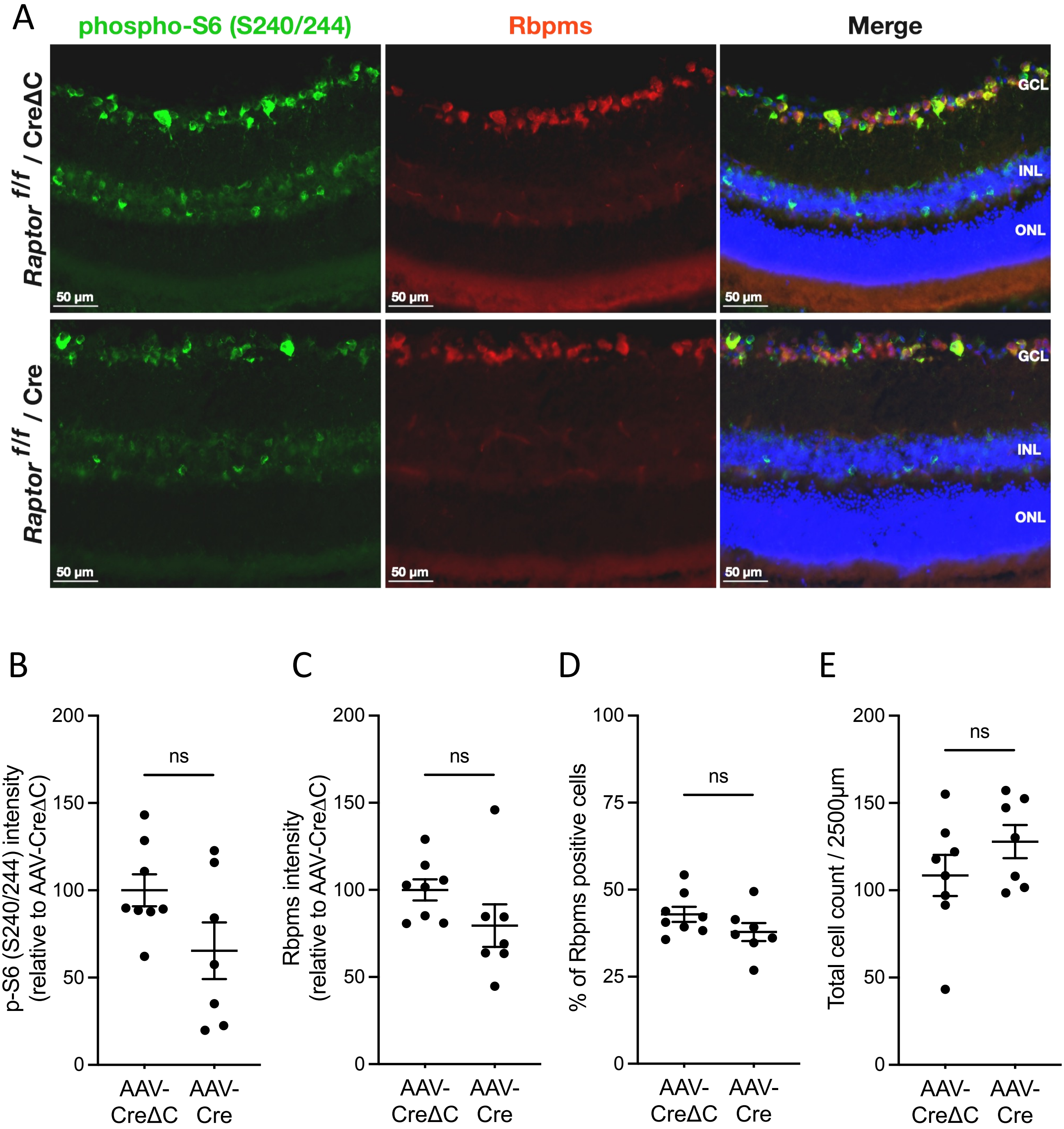

Supplemental Figure S9
